# Machine learning based anti-cancer drug response prediction and search for predictor genes using cancer cell line gene expression

**DOI:** 10.5808/gi.20076

**Published:** 2021-03-26

**Authors:** Kexin Qiu, JoongHo Lee, HanByeol Kim, Seokhyun Yoon, Keunsoo Kang

**Affiliations:** 1 Department of Computer Science, Dankook University, Yongin 16890, Korea; 2 Department of Electronics and Electrical Engineering, Dankook University, Yongin 16890, Korea; 3 Department of Microbiology, Dankook University, Cheonan 31116, Korea

**Keywords:** cell line gene expression data, drug response prediction, machine learning, predictor genes

## Abstract

Although many models have been proposed to accurately predict the response of drugs in cell lines recent years, understanding the genome related to drug response is also the key for completing oncology precision medicine. In this paper, based on the cancer cell line gene expression and the drug response data, we established a reliable and accurate drug response prediction model and found predictor genes for some drugs of interest. To this end, we first performed pre-selection of genes based on the Pearson correlation coefficient and then used ElasticNet regression model for drug response prediction and fine gene selection. To find more reliable set of predictor genes, we performed regression twice for each drug, one with IC_50_ and the other with area under the curve (AUC) (or activity area). For the 12 drugs we tested, the predictive performance in terms of Pearson correlation coefficient exceeded 0.6 and the highest one was 17-AAG for which Pearson correlation coefficient was 0.811 for IC_50_ and 0.81 for AUC. We identify common predictor genes for IC_50_ and AUC, with which the performance was similar to those with genes separately found for IC_50_ and AUC, but with much smaller number of predictor genes. By using only common predictor genes, the highest performance was AZD6244 (0.8016 for IC_50_, 0.7945 for AUC) with 321 predictor genes.

## Introduction

Cancer is one of main causes of death worldwide. Anti-cancer drug therapy is an important part of cancer treatment and an effective use of them can prolong patient’s survival. According to many clinical data, patients with the same cancer have quite different response to the same treatment or the same drugs due to genomic specificity. Recently, targeted anti-cancer therapy [1,2] considering gene-specific effects has been proposed as a new cancer therapy. In order to develop specific targeted therapy for cancer patients in clinical treatment, many clinical trials are required. However, there are many obstacles such as sample limitations, complicated operations, high environmental requirements, and high cost, which far from meeting the demand.

With the rapid development of artificial intelligence, many machine learning based drug response prediction models were proposed utilizing genomic information and anti-cancer drug response data. In 2011. Riddick et al. [3] used the random forest algorithm to establish a regression model of drug response, and successfully predicted the drug response of 19 breast cancer and seven glioma cell lines, which was advanced to other methods such as based on differential gene expression. In 2014, Geeleher et al. [4] used Ridge regression based on baseline gene expression levels and in vitro drug sensitivity in cell lines to establish a regression model and used it to predict clinical drug response. On the other hand, some studies have shown that the structural similarity between drugs may have similar response to cancer cell lines that have similar gene expression profile [5-7]. Specifically, Shivakumar and Krauthammer [8] reported that the similarity between drugs is useful to predict the drug response. Based on this research background, we designed an improved drug response prediction model based on cancer genomics data and explored the predictor genes possibly related to the drug response.

## Methods

### Data

The data used in this work is from Genomics of Drug Sensitivity in Cancer (GDSC) [9] which was developed by the Sanger Research Institute in the United Kingdom. We considered 12 drugs and gene expression data for 1,000 human cancer cell lines. The drug response indicators used were the half maximal inhibitory concentration (IC_50_) and the area under the curve (AUC) [10]. The former is the concentration at which the compound reaches 50% reduction in cell viability and the latter is the area under the fitted dose response curve. Biologically, the smaller the IC_50_ and AUC, the greater the response of the cancer cells to the drug.

### Method

**Fig. 1.**
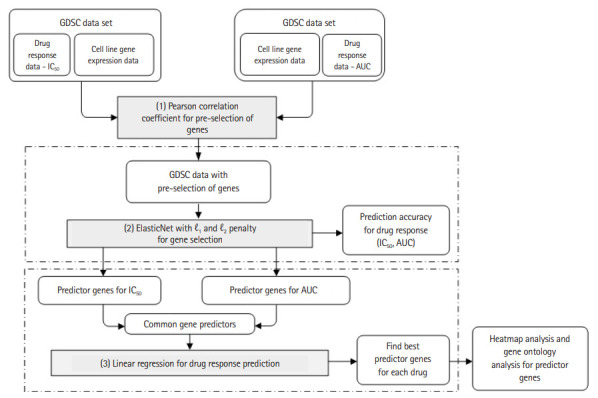
Experimental workflow. GDSC, Genomics of Drug Sensitivity in Cancer; AUC, area under the curve.

Based on the gene expression data of the cancer cell lines and the two types of response indicators, we used a machine learning algorithm to construct a drug response prediction model. We first pre-selected genes based on the p-value of Pearson correlation coefficients [11] and then used ElasticNet to predict drug response and to further select the predictor genes among the pre-selected ones. Specifically, we performed ElasticNet regression separately on the two response values, from which common predictor genes were identified. These common genes were used again to predict drug response hoping that the prediction performance is better than, or at least similar to, those obtained separately for the two response indicators. To confirm biological significance of predictor genes, we provide heatmap and gene ontology analysis results. Fig. 1 shows the entire experimental workflow.

#### Preprocessing

Before processing the data, we took logarithm on IC_50_ and normalized the cell line gene expression data using the robust multichip average [12].

#### Feature selection based on Pearson correlation coefficient

For some drugs, there are thousands of genes in the gene expression data, but not many genes have strong correlation with the drug responses. Therefore, it is very important to pre-select the relevant genes first. Although ElasticNet has capability for gene selection, it is subject to data dependency and/or batch effect and, sometimes, it ignores genes that are really important to predict drug responses. In this paper, to overcome such problem, we used two-step gene selection, where we first used the Pearson correlation coefficient to pre-select genes and then applied ElasticNet to fine select the predictor genes. In particular, we used p-value of Pearson correlation coefficient between the drug response and the expression of each gene, with which genes with p = 0.05 or less were selected in the first feature selection.

#### ElasticNet-based feature selection and drug response prediction

**Fig. 2.**
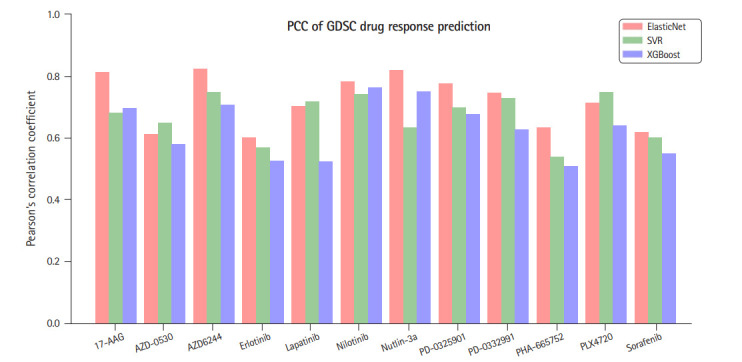
A comparison of three regression methods in terms of Pearson’s correlation coefficients (PCC) between the predicted IC_50_ and the measured ones. GDSC, Genomics of Drug Sensitivity in Cancer.

ElasticNet [13] is a linear regression model trained with both ℓ_1_ and ℓ_2_ regularization. It is useful when there are so many features that are correlated with one another. In our data, the number of features (genes) is much larger than the number of samples and the prediction might be subject to overfit. Hence, to appropriately select genes and to suppress generalization error, we used ElasticNet to predict the drug response. The ElasticNet was selected based on the preliminary experiments where we compared ElasticNet with two well-known models, SVR [14] and Xgboost [15]. The former can be configured to a non-linear regressor by using various kernel functions and we used radial basis function kernel and the latter is an improved version of decision tree based gradient boosting algorithm. The two algorithms were shown to perform good for many applications, while, according to our preliminary experiments, they seem to have higher overfit than ElasticNet as the numbers of predictor genes that are common for the two response indicators were smaller than that for the ElasticNet. Fig. 2 summarizes the comparison for the 12 drugs in terms of Pearson correlation coefficients between the predicted IC_50_ and the measured ones.

## Results

### Prediction of IC_50_ and AUC

In the first experiment, we predict the two drug response indicators, IC_50_ and AUC separately. In ElasticNet, there are two key hyper parameters, a.k.a. the penalty weight α and the relative weight of ℓ_1_ penalty λ, where α is an arbitrary positive real while 0 ≤ λ ≤ 1. λ = 0 corresponds to the Ridge regression, where we have only ℓ_2_ penalty while λ=1 corresponds to LASSO regression where we have ℓ_1_ penalty only. These two hyper parameters must be optimized to achieve the best performance. To this end, we performed grid search for a set of combinations (α, λ). Through this, the best performance for drug response prediction were obtained for the 12 drugs as summarized in Table 1.

For all the 12 drugs, the correlation coefficient between the estimated IC_50_ and the true ones were higher than 0.65, where three of them were reached 0.8, e.g., 0.823 for AZD6244, 0.819 for Nutlin-3a and 0.811 for 17-AAG. Similar performances were also observed for AUC, where 17-AAG and nilotinib showed correlation coefficient exceeding 0.8. The results seem to be statistically significant as the p-value for the correlation coefficient of each drug was less than 0.01.

Of note, not only the number of genes to obtain the optimal predictive performance were quite different for each drug but also the gene sets for the two response indicators of the same drugs were only partly overlapped. The latter suggest us that there might exist dependency on the response indicators and it would be interesting to check the prediction performance using the common predictors. Hopefully, they will be more reliable predictors of the practical drug responses.

### Drug response prediction based on common predictor genes

In the previous experiments, we found the predictor genes separately for the two response indicators and it is also interesting to evaluate the performance when using only common genes. It could be a more stable group of predictor genes for drug response. To find the predictor genes that are commonly effective in the two response indicators, the relative weights of the ℓ_1_ and ℓ_2_ of the ElasticNet were fixed to 0.5. Then we adjusted α to make the number of selected genes for the two response indicators are similar to each other and then took the intersection of them to obtain the common predictors to be used for the drug response prediction. The results are summarized in Supplementary Fig. 1 and the scatter plots of the predicted versus the true responses were shown in Supplementary Fig. 2 for IC_50_ and 3 for AUC.

As the number of common genes increases, the predictive performance for each response indicators changed similarly, but it is confirmed that the performances were saturated or slightly decreased after reach the peak. According to the trend of the prediction accuracy curve, we found the points at which the performance was the best for both the drug response indicators simultaneously. The results are summarized in Table 2, where the Pearson correlation coefficients for IC_50_ and AUC of six drugs were higher than 0.7 only with 200 predictor genes. Comparing with the results in Table 1 for separate predictors for each response indicator, it can conclude that even with only those common predictor genes, one can have similar predictive performance suggesting that these genes are more reliable predictors on the two response indicators and are closely related to the underlying biological mechanism that governs the drug response. For comparison, we provided the performance of the drug responses in the literature for the same GDSC dataset in the last column of Table 2.

**Fig. 3.**
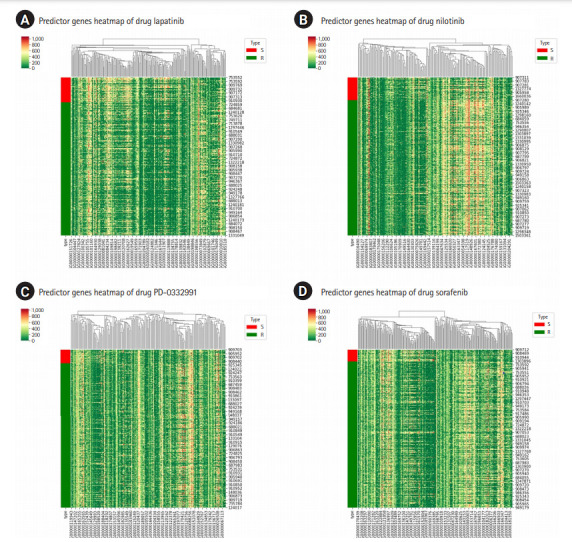
Heatmap for the predictor genes of the four selected drugs: for lapatinib (A), for nilotinib (B), for PD-0332991 (C), and for sorafenib (D). The type abbreviation S stands for “sensitive” and R for “resistant.”

GSDC data set also provides binary indicator of drug response, with which the cell lines are labelled as either “sensitive (S)” or “resistant (R)” to a specific drug. And it would be interesting if the two groups show non-negligible difference in the expression of the predictor genes or not. Fig. 3 shows the heatmap for the predictor genes for four drugs, where we can identify the differences in their expressions between the two group and can qualitatively judge the effectiveness of the predictor genes we found. The heatmap analysis [16] shows that the predictor genes can also distinguish the drug sensitivity of cell lines to a certain extent, even though it is not our focus in this work. Rather, it would be more interesting to check what biological processes these genes are involved in response to a certain drug treatment.

### Notes on biological implication of the predictor genes

To show the biological implication of the drug response, we used Metascape [17] to perform gene enrichment analysis. The predictor genes for the 12 drugs were listed in Supplementary Table 1 and the results of gene enrichment analysis for the 12 drugs are shown in Supplementary Table 2. Through the enrichment analysis of predictor genes, we found various pathways that were mostly related to cancer, such as cell proliferation and developmental process. For example, the negative regulation of cell population proliferation (GO:0008285) is a process that stops, prevents, or reduces the rate or extent of cell proliferation [18]. If predictor genes of drug found by machine learning are in this pathway, this drug may be effective for cancer.

Of note, AZD6244 is an inhibitor of the MAPK cascade [19]. The predictor genes we found were confirmed to be related to the regulation of the MAPK cascade through the enrichment analysis. Nutlin-3a is known to be an inhibitor of the MDM2-p53 (TP53) interaction [20]. The first significant pathway of the predictor genes appeared to be the p53 downstream pathway. It can be seen that some genes that are important to predict drug response are related to the mechanism of drug action. For example, of NQO1 found to be one of the predictor genes of 17-AAG, the overexpression was known to increase the sensitivity to the drug 17-AAG [21]. Among the predictor genes of Nutlin-3a, the regulation of HIPK2 determines the response of tumor cells to the p53 activating drug Nutlin-3a [22]. platelet-derived growth factor receptor A, one of the predictor genes of PD-0332991, is known to play an important role in cell signaling pathways that affect cell growth and differentiation and are associated with an array of clinically significant neoplasms [23]. For other drugs, it may be a new mechanism of action for drugs which is not yet known.

## Discussion

Although the model proposed in this study shows good predictive performance for GDSC, there are still some limitations. First, the characteristic of cancer cell line may be quite different from the in vivo cancers and it should be verified whether this will be effective in clinical trial. Second, we perform drugs response prediction mainly based on gene expression data. While, the response of drugs is not only related to gene expression levels, but also to structural variations such as gene mutations. Therefore, more study is required to utilize such information and integrate them into the model to improve the predictive power.

Cancer is one of the leading causes of death worldwide. If one can find a new treatment by accurately predicting drug response, the probability of recovery will also be increased. Although there are still huddles to overcome in drug response prediction, advances in machine learning techniques will make it possible to introduce new ideas for drug response prediction that can provide accurate drug treatments and make it practical for clinicians and non-experts.

## Figures and Tables

**Table 1.** Comparisons of the PCC between the estimated response and the true value for the 12 drugs in GDSC

Drug name	Predict IC_50_		Predict AUC	
	No. of features	PCC	No. of features	PCC
17-AAG	566	0.811	520	0.81
AZD-0530	262	0.612	214	0.702
AZD6244	570	0.823	551	0.792
Erlotinib	253	0.603	222	0.60
Lapatinib	261	0.698	213	0.625
Nilotinib	475	0.782	340	0.839
Nutlin-3a	475	0.819	310	0.783
PD-0325901	570	0.775	520	0.742
PD-0332991	527	0.743	432	0.671
PHA-665752	224	0.635	155	0.522
PLX4720	499	0.715	348	0.705
Sorafenib	297	0.619	248	0.647

PCC, Pearson’s correlation coefficient; GDSC, Genomics of Drug Sensitivity in Cancer; AUC, area under the curve.

**Table 2.** Comparisons of the PCC of the predicted IC_50_ and AUC with those reported in literature [6]

Drug name	No. of features	PCC of the predict IC_50_	PCC of the predict AUC	Existing prediction results of IC_50_ [6]
17-AAG	260	0.795	0.785	-
AZD-0530	80	0.547	0.591	0.58
AZD6244	321	0.8016	0.7945	0.6
Erlotinib	43	0.505	0.562	0.590
Lapatinib	229	0.588	0.61	0.585
Nilotinib	184	0.745	0.799	-
Nutlin-3a	198	0.764	0.742	-
PD-0325901	234	0.742	0.728	0.8
PD-0332991	195	0.707	0.688	-
PHA-665752	48	0.468	0.359	0.35
PLX4720	171	0.643	0.654	0.57
Sorafenib	244	0.595	0.583	0.38

The results show that the proposed method performs better for most of the drugs we tested than other methods. PCC, Pearson’s correlation coefficient; AUC, area under the curve.

## Supplementary Materials

Supplemental Figure 1. Pearson correlation coefficients between the true response and the predicted ones with different number of selected genes for the two response indicators.

Supplemental Figure 2. Scatter plot of the true and predicted IC_50_ for the 12 drugs in GDSC

Supplemental Figure 3. Scatter plot of the true and predicted AUC for the 12 drugs in GDSC.

Supplemental Table 1. List of Predictor genes for the 12 drugs in GDSC.

Supplemental Table 2. Gene ontology analysis results on the predictor genes for the 12 drugs in GDSC. We used Metascape to perform gene enrichment analysis to determine the gene functions, related pathways and their enrichment levels of common predictor genes we found for 12 drugs. (The number of common predictor genes for each drug is shown in Table 2)
