# Supplementary material for: Machine learning based anti-cancer drug response prediction and search for predictor genes using cancer cell line gene expression
Source: Genomics Inform. 2021 Mar 26;19(1):e10. doi: 10.5808/gi.20076 (PMC8042299; doi:10.5808/gi.20076)
Supplement: Supplemental Figure 1. — Pearson correlation coefficients between the true response and the predicted ones with different number of selected genes for the two response indicators. [file gi-20076suppl1.docx]

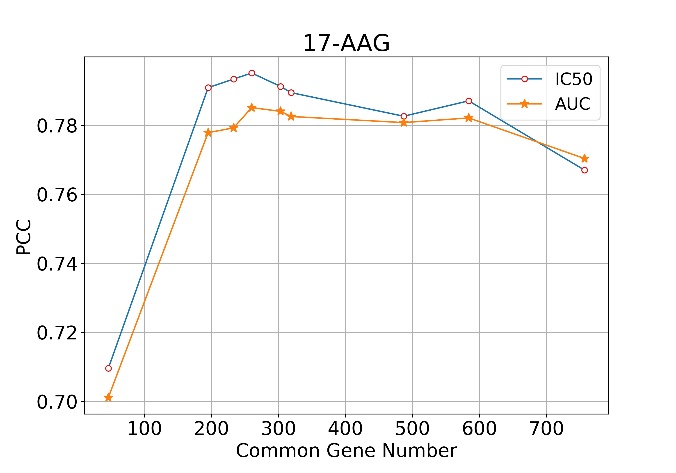

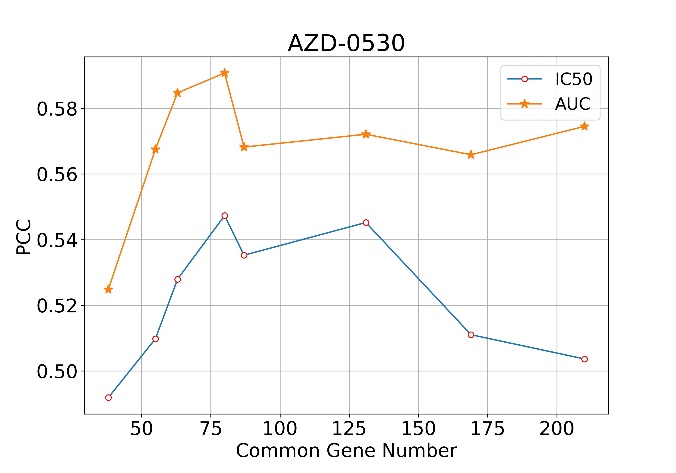


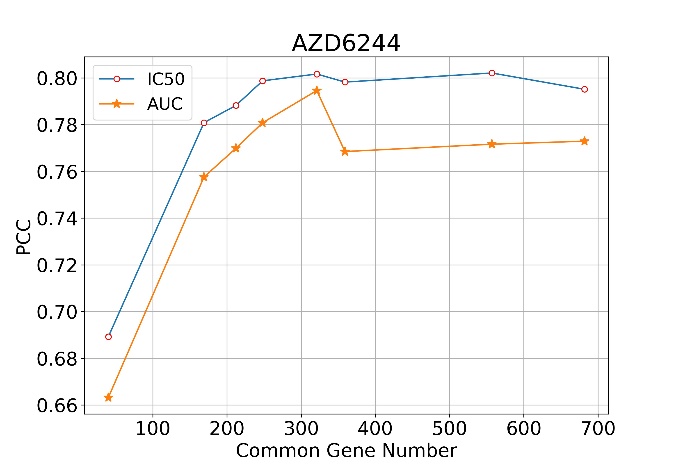

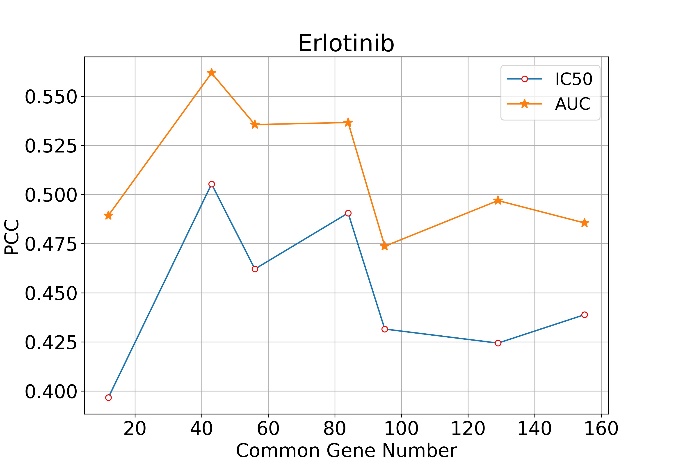


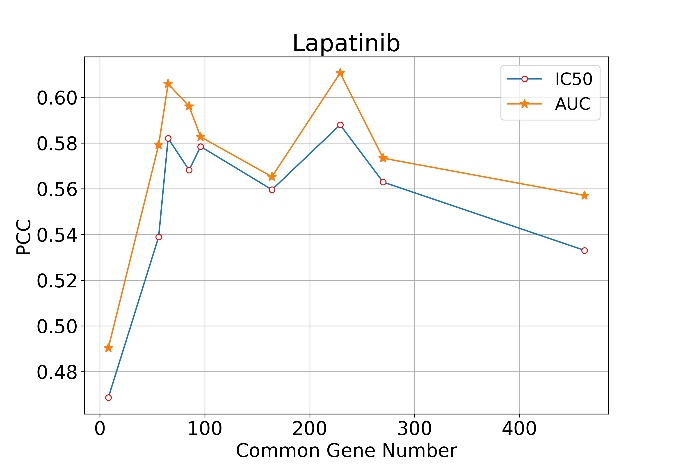

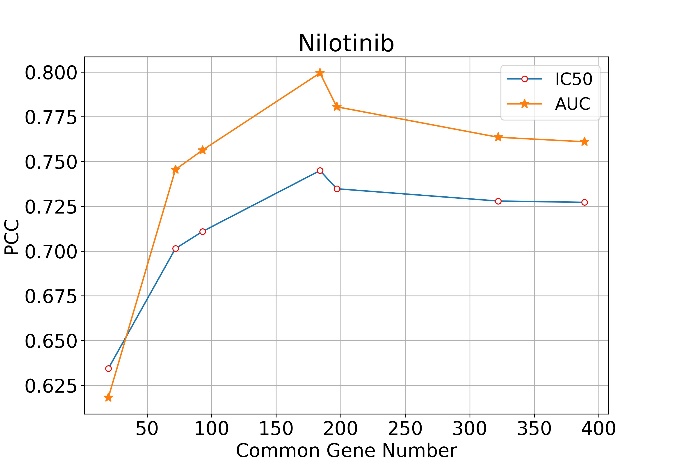


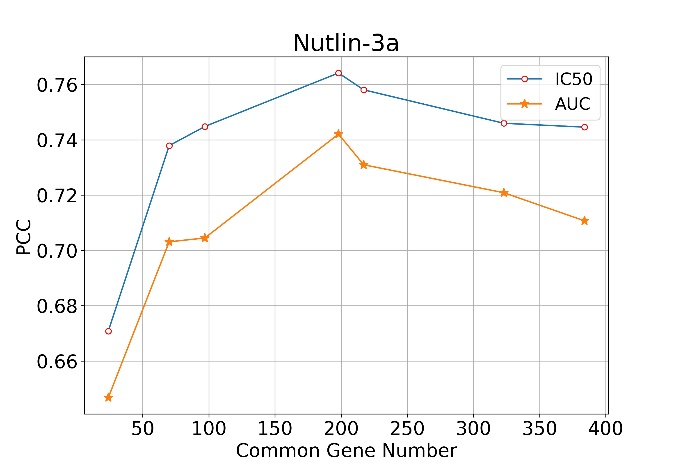

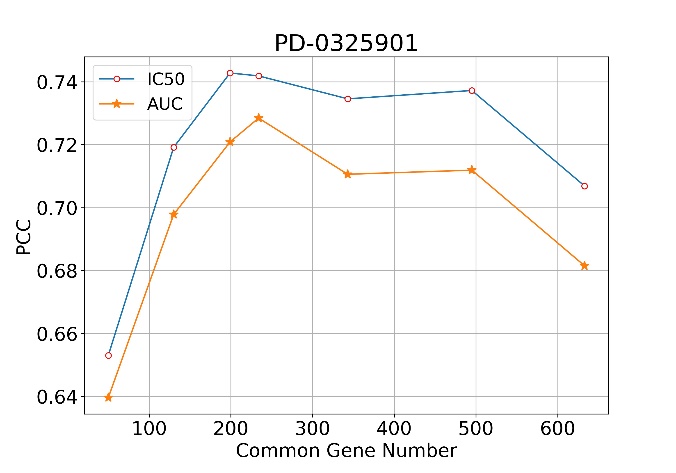


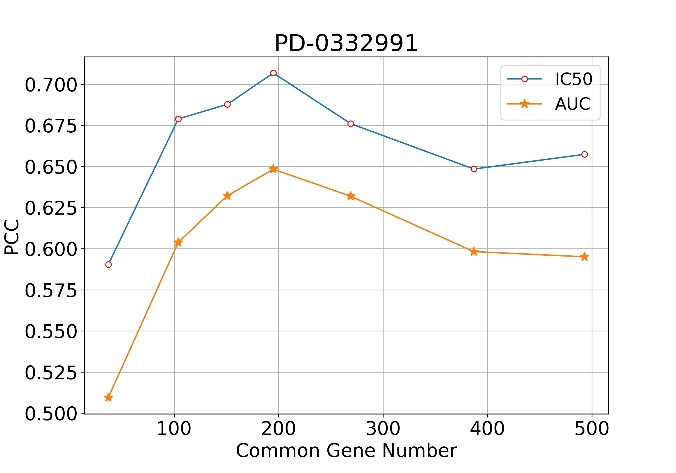

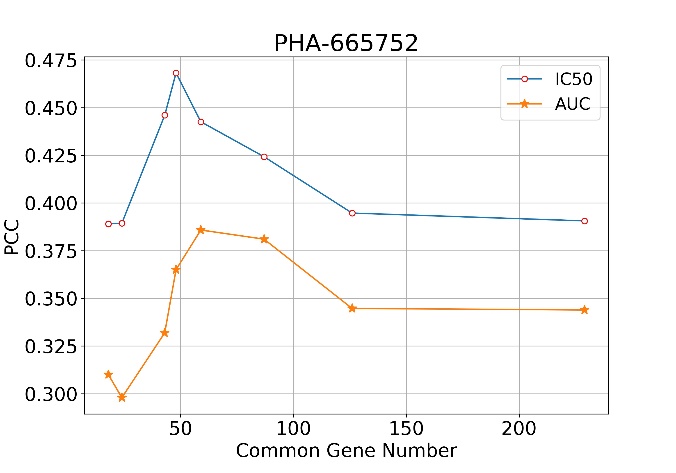


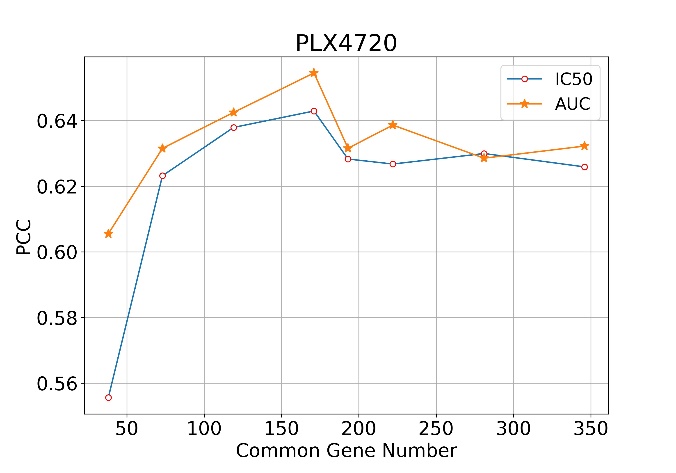

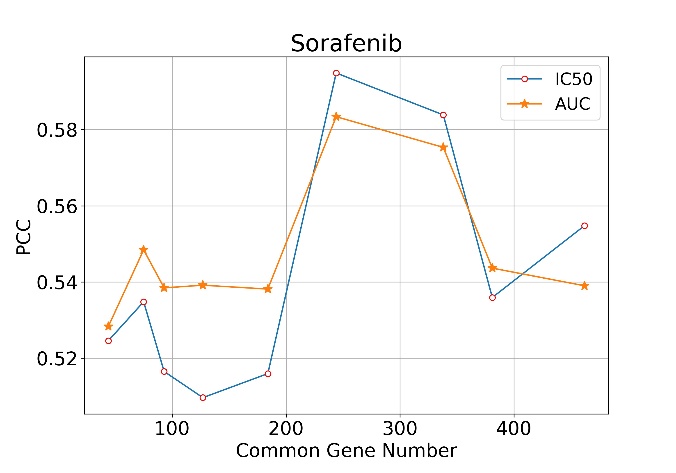


**Supplemental Figure 1** Pearson correlation coefficients between the true response and the predicted ones with different number of selected genes for the two response indicators.
