# Supplementary figures and images for: Machine learning based anti-cancer drug response prediction and search for predictor genes using cancer cell line gene expression
Source: Genomics Inform. 2021 Mar 26;19(1):e10. doi: 10.5808/gi.20076 (PMC8042299; doi:10.5808/gi.20076)

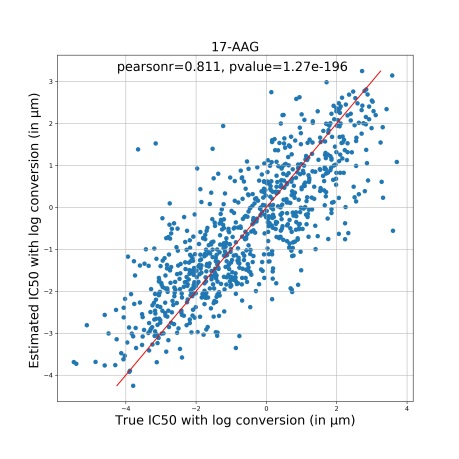

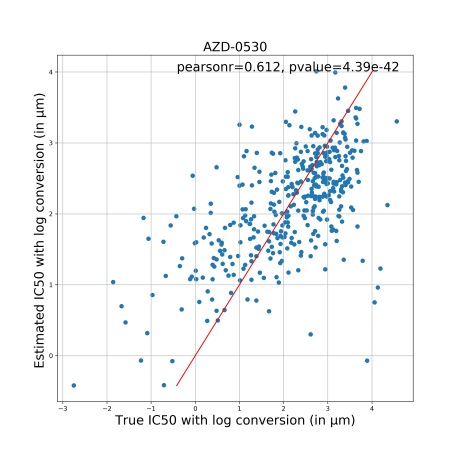

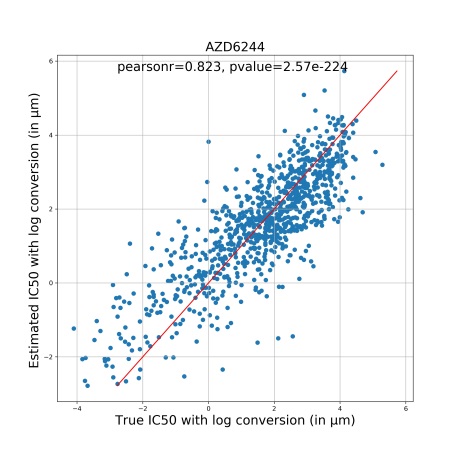


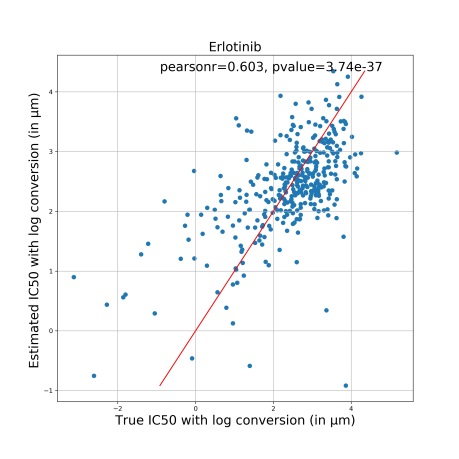

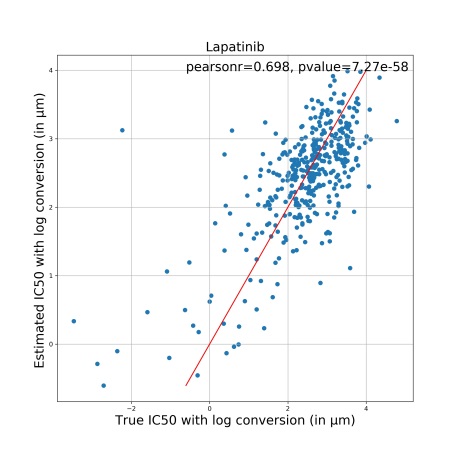

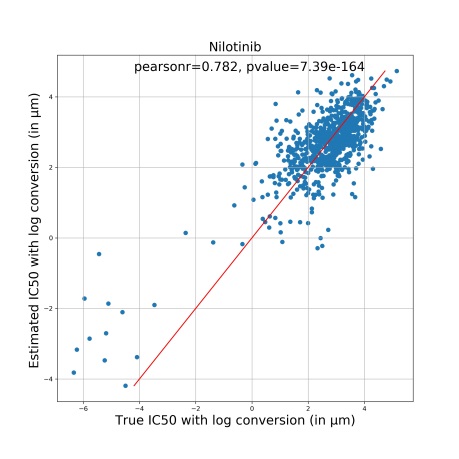


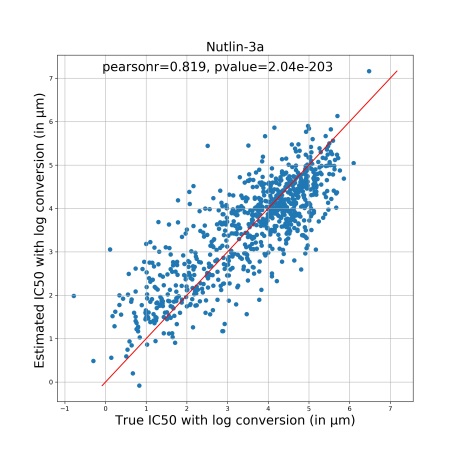

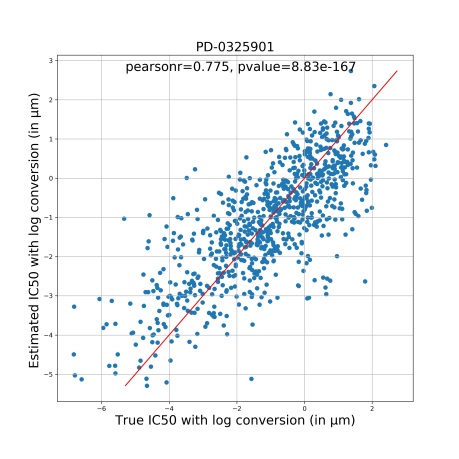

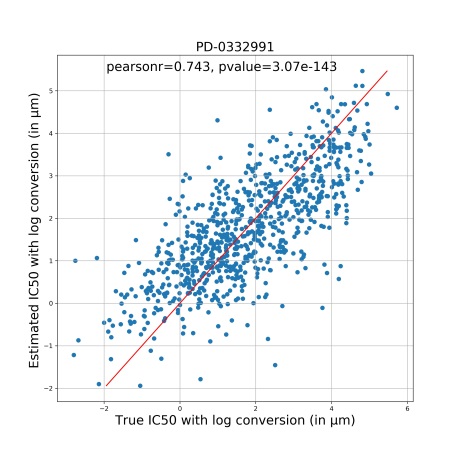


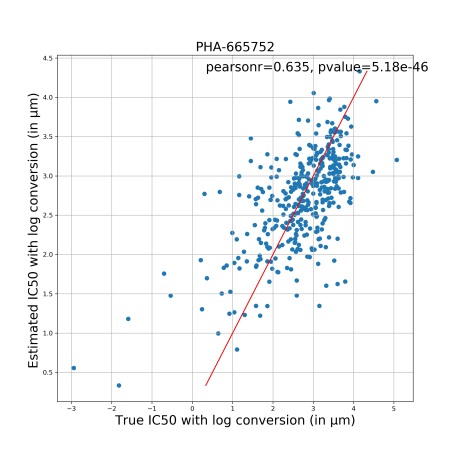

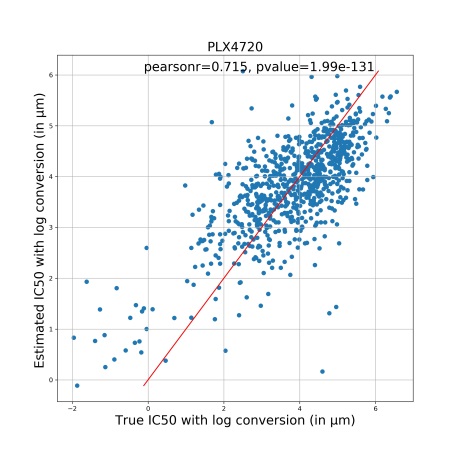

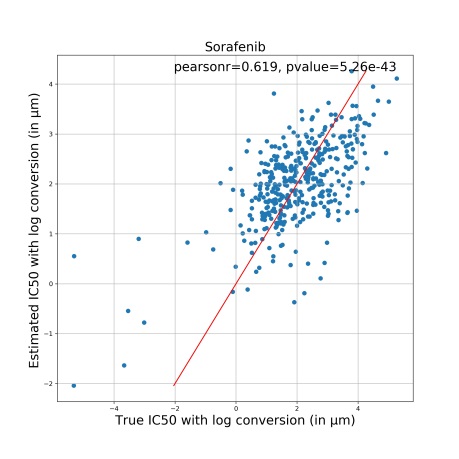


**Supplemental Figure 2** Scatter plot of the true and predicted IC50 for the 12 drugs in GDSC

Supplement: Supplemental Figure 2. — Scatter plot of the true and predicted IC50 for the 12 drugs in GDSC [file gi-20076suppl2.docx]

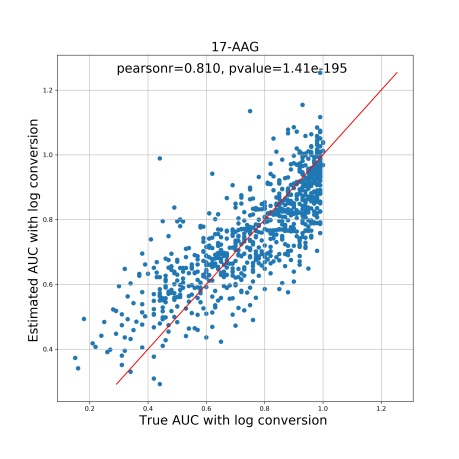

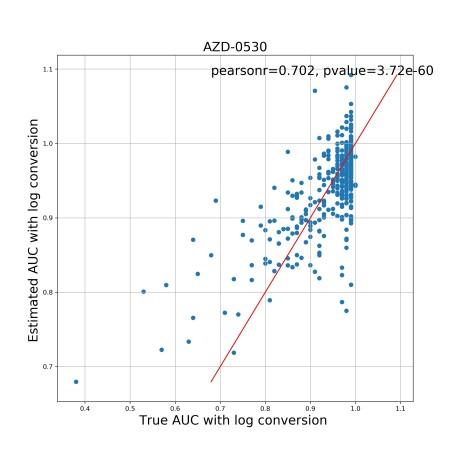

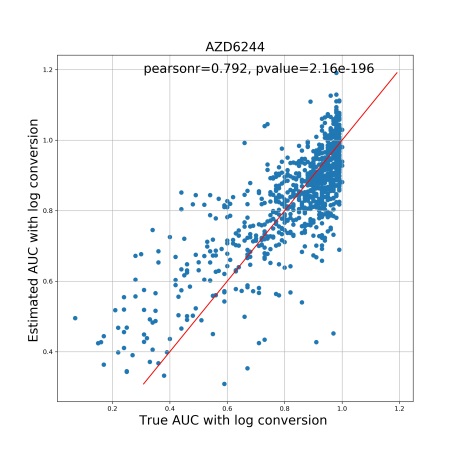


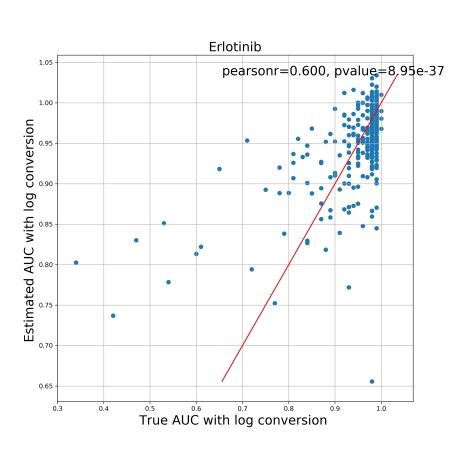

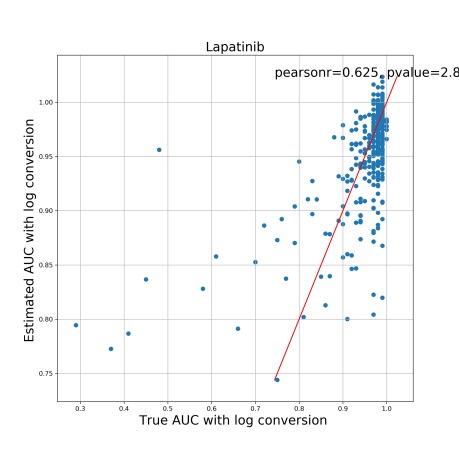

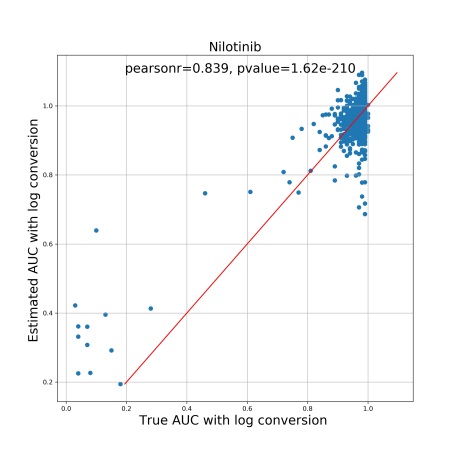


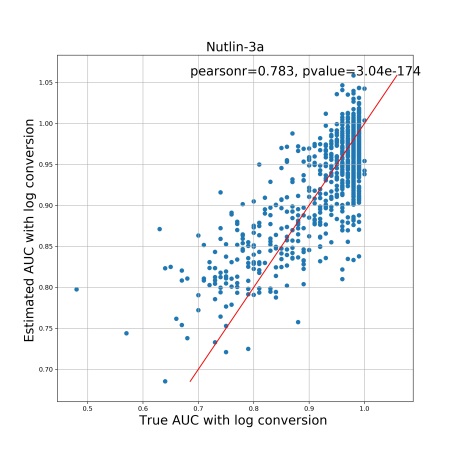

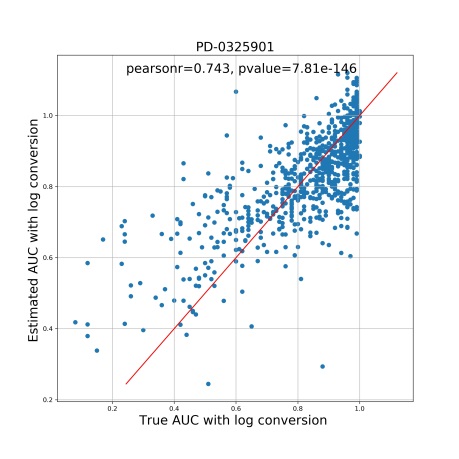

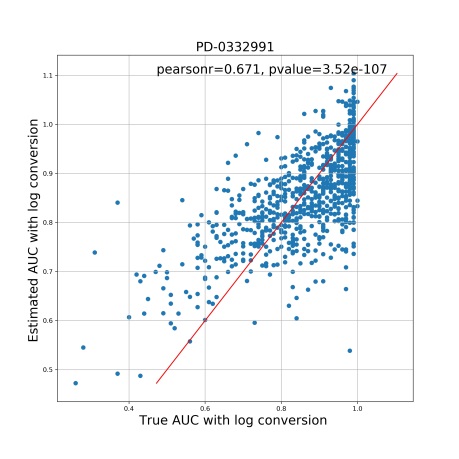


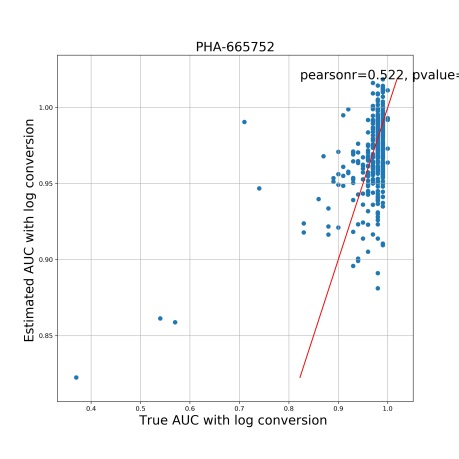

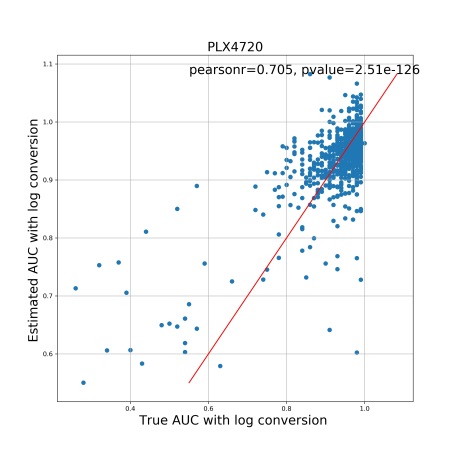

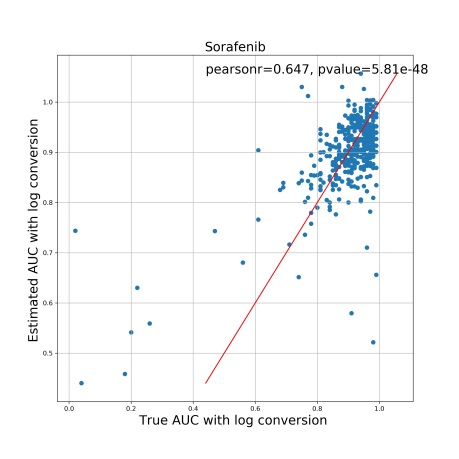


**Supplemental Figure 3** Scatter plot of the true and predicted AUC for the 12 drugs in GDSC.

Supplement: Supplemental Figure 3. — Scatter plot of the true and predicted AUC for the 12 drugs in GDSC. [file gi-20076suppl3.docx]
