# Supplementary material for: Machine learning based anti-cancer drug response prediction and search for predictor genes using cancer cell line gene expression
Source: Genomics Inform. 2021 Mar 26;19(1):e10. doi: 10.5808/gi.20076 (PMC8042299; doi:10.5808/gi.20076)
Supplement: Supplemental Table 1. — List of Predictor genes for the 12 drugs in GDSC. [file gi-20076suppl4.docx]

**Supplemental Table 1** List of Predictor genes for the 12 drugs in GDSC.

| Predictor genes list for drug 17-AAG |
| --- |
| *BMP2, POGLUT3, PROCR, KLF9, F12, AOX1, CMTM3, PEA15, FAM107B, TWIST1, REC8, TGM2, CD200, MEIS1, ANPEP, SH3RF3, COL6A3, CLDN4, KCNMB4, RASGEF1A, NUP62CL, SERPINI1, ATP8B1, KCNN4, TMEM170B, MANSC1, SAA1, ABCC3, PROM1, FTH1, VEGFC, KRT86, CST6, DLK2, ARHGAP29, CCL2, ZNF268, ERRFI1, GATM, COL1A2, COL2A1, PAQR8, RAB10, SERPINE2, SOX8, PLP2, CCDC167, CDCA7L, OXCT1, MMP13, GK, THSD7A, GLIPR1, TIMP1, DDR2, ZNF83, GLRB, PLS3, AVPI1, PLCE1, ADGRA2, PLA2G4A, FGGY, MGST1, BEX4, DUSP23, MICALL2, CYFIP2, PCP4, FBXL16, YPEL3, LAMA4, ANXA1, TFAP2B, PAPSS2, ABCB1, SLC30A1, ASPHD1, SEMA3A, GNG4, MND1, G0S2, ICAM4, SDC1, BAMBI, RNF182, SH3GL3, QPCT, SLC35E3, AK5, GAS6, GSTT1, CACNA2D3, GYPC, HYI, PSMB8, LGALS1, NPDC1, ALDH3A1, EFNA5, KRT7, NRN1, LRCH2, SCG2, PRKD1, UCA1, PFKP, CD55, C19orf33, SGK223, FGFBP1, KRTAP23, FKBP14, ENAH, SDCBP, CD99, LIPT2, CD1D, TFDP2, DUSP6, COL7A1, LRFN5, TFAP2C, SLC43A1, MYO6, IFI44L, FBLN5, AKR1C2, TMEM44, TP53I3, NIBAN1, RIMS2, WHAMMP3 , TNNT1, GSPT2, SERPINA1, APBB1IP, TRIM31, KRTCAP3, CLIC6, PPP1R3C, CAPG, PLAAT1, CSRP2, ZNF204P, OGFRL1, GASK1B, NECTIN3, NUPR1, NQO1, PNMA8A, HLAC, GLI3, TCIM, ZNF738, GPNMB, IGFBP2, TMEM45A, ZNF114, EFEMP2, RRAGD, COLCA2, EYA4, ITM2A, DUSP1, PTHLH, SERPINB1, MIR205HG, G6PD, EVA1C, HLADRA, NAV2, PTX3, NAV3, ANK3, CHCHD7, SAMD13, ST6GALNAC2, HSPB8, HMOX1, EXD2, COL4A2, EFEMP1, S100A11, KIF15, CCN3, PYCARD, HNMT, ADCYAP1, FAT3, HLADPA1, RAB34, IGFBP6, MZT2A, MAGEH1, TNC, COLEC10, AKR1C3, PRKCD, ATAD1, TSPAN1, LY6D, C3orf14, TSPAN6, HTRA1, NOSTRIN, FUNDC1, ALDH1A1, IFIT2, GAA, CDH1, DHRS7, DUSP4, ACSL3, DNAJA4, PRSS23, FILIP1L, GTSF1, CAVIN3, SC5D, SMARCA2, TREM1, CCR7, C15orf48, FOXC1, PMP22, MYO1E, SLC47A2, COL18A1, EMB, KRT14, SMIM3, TSC22D1, IFI27, BABAM2, NTN4, TRABD2A, PDE9A, PLLP, SMIM6, NTF3, MFSD12, ZNF667AS1, MXRA5, PTEN, DKK1, BIN1, SRPX2, OPLAH, EGFR, ATL3, APOBEC3B, RUNDC3B, CMBL, ENDOD1, CDCP1, IRX3, ARHGEF10, CALD1, PRRG4* |

| Predictor genes list for drug Nilotinib |
| --- |
| *GPNMB, PHGDH, TACSTD2, GPR65, PROK2, CPPED1, ALDH1A3, BEX3, GAREM1, LIMA1, ENPEP, GDF15, BCAS3, UBASH3B, SNX19, ANK1, C1QL1, SPATS2L, PARP8, NXF3, SNX7, LAYN, HLA-C, BASP1, FOXC1, S100A14, CPED1, SIX4, RP11-388M20.9, S100A2, CSMD1, IGLL1, PERP, CAV1, C1QTNF4, TM4SF1, PRSS23, GADD45B, EMP1, MS4A3, RAB27A, STC1, RAB33A, CCDC173, GNG12, SPA17, TUBAL3, 43891, FERMT3, ANTXR2, C19orf33, KIF21A, MTO1, BIN1, FADS1, SCML1, MGST3, CA8, RASGEF1A, ACBD3, SDC4, ZDHHC14, FUT7, PLIN2, FOS, RHOH, PIM1, FAT1, APP, C1orf186, BST2, RASGRP2, FAM162B, AEBP1, C15orf48, ENC1, ARNTL2, WASF1, TDRD9, TSPAN13, PABPC4L, CST7, TSC22D3, EMP2, SGCG, BTG3, TRPC1, GPC4, STAT4, TBXA2R, CNN3, C3orf14, TMEM154, BBS12, GPX8, LCP1, KCNN4, CLCA1, ZBED8, SAMSN1/LOC388813, ALOX5AP, PIR, DUSP1, CLEC2B, NKD2, S100A13, FZD6, CLDN1, TESC, SEC31B, KAT2B, EN2, PPDPF, TRIM13, LGMN, SPRR2D, LPXN, PCDHB5, FAR2, MLF1, GTSF1, SELL, ITPRIPL2, ST6GALNAC2, SLAIN1, FUCA1, CISH, SOCS2, CYBA, ANKRD27, MGLL, ARHGEF5, PYCARD, MAGED2, ADM, CSRP2, C20orf197, ATM, CD52, GAS6, TMTC1, DENND3, B3GNT2, COL15A1, VIM, PSTPIP2, TRIM22, HYI, MFHAS1, S100A10, SCG2, PKN1, OSCAR, CTSF, ARHGAP10, DOCK2, C9orf64, CGAS, RGS10, ICAM3, PRSS57, SQOR, AXL, ZBTB16, LOC339803, ANKRD55, DNAJC12, CFAP161, MOXD1, CBR3, RTL8A, NGEF, LITAF, KRT7, HMGN5, HTRA1, S100A4, SELENOP, FAM71E1, FHIT, CAMK2D, SOX9, PLK2, TPD52L1* |

| Predictor genes list for drug Lapatinib |
| --- |
| *ATP8B1, PMAIP1, LGALS3, SV2B, GALNT7, LOXL1, GCLC, GPR183, PACSIN3, ATP6V0A4, ZNF789, SUSD2, BDKRB1, EPCAM, MSX2, DOCK11, KLHDC7A, TUBA4A, PTHLH, PLAU, CDA, S1PR5, FERMT1, SPINK1, PNMA2, TENT5B, TST, CXCL2, ST3GAL1, RTN1, SERPINB7, S100A8, HEXB, SNX7, CLMN, MATN2, FOXC1, SLC16A1, STX3, C16orf74, DKK1, S100A14, PALLD, INHBB, GRAMD2B, MTCL1, P4HA2, DHRS2, OPLAH, SLC10A4, PERP, UPK1B, WDR54, PRR15, RAB20, SLC40A1, PTCH1, SPRR1A, IGFBP6, ST6GAL1, DLL3, TRPS1, PSMD5, CCDC88A, S100A16, LCN2, SMAD3, SRPX2, SDCBP2, KRT86, STC2, C19orf33, CXCL16, TFF1, IRX3, TUBB2B, FYN, TPD52L1, ZNF195, BPIFA1, SLC2A1, FGFBP1, CLDN4, CTSO, ANXA10, CST6, ACOT9, RORA, BAHCC1, AEBP1, NFKBIZ, KYNU, SPHK1, CYP24A1, IL1A, SERPINB6, KLF5, FRMD6, ACSL1, PLEKHO1, MGST1, CXCR4, RHBDL2, CXCL8, AC093840.1, BTK, CTSV, CYP1B1, PHF10, IFI27, TSC22D3, GAMT, MB, ME1, FBXO32, DUSP5, JAKMIP2, TOX, MPZL2, KCNS3, EREG, CD82, SLC16A3, AGR2, SCEL, THBS1, UGDH, OASL, FZD6, CLDN1, KLK8, C3, TESC, PLEK2, FOXQ1, CTSZ, CAV2, PPDPF, MAGEC1, IGF2BP2, LPXN, ZNF773, RFXAP, NNT, C2CD4A, SELENOM, PTGES, TGFBI, ZG16B, HEBP1, FABP4, FGGY, ST6GALNAC2, ALDH3A1, ABCC5, LOXL4, LAMB2, CYP4F11, DSC2, NOL4, APLP2, EFNA1, ARHGEF5, SERPINB3, EBF1, FAM184A, HTATIP2, AMIGO2, PHACTR1, GABRP, CTSH,* *MMP28, PDLIM1, TNFRSF12A, ASRGL1, PNMA8A, CDKN2C, S100A9, SEMA3B, ZNF204P, HPDL, GPRC5C, HSPA1A, HOXB7, CXXC4, ERBB3, RNF128, SYBU, HERC6, CRISP3, PAPSS2, KRT80, CD9, COL7A1, KRT5, ITGA2, AHR, GPC2, SLPI, APOL1, PPL, F3, ITGA6, KRT15, RRAGD, BAIAP2L1, BSPRY, CDS1, PYGL, DEAF1, PLEC, TRIP6, THSD7A, ERRFI1, HMGB2, DNAJA4, ETHE1, ANXA4, RBMS1, CYFIP2, PLS1, MYCBP2, SNAI2, TES, CYP1A1, CORO1A, PLK2, ANKRD22, BEX1* |

| Predictor genes list for drug AZD6244 |
| --- |
| *MSN, LRRC61, CD109, C4orf48, UCHL1, TNFRSF19, DEPDC1, BASP1, MN1, BEX2, DET1, CAPG, MECOM, LRP3, PRSS23, NRIP3, RPS6KA2, SLFN12, CHST7, CDC42EP3, BMP7, CDK6, SERPINA3, NT5E, SOWAHC, HAT1, ARG2, TM4SF4, MX2, SLC16A4, KISS1, C15orf39, ELAVL2, TMPRSS3, ENC1, GJB1, ZNF630, SLC16A6, CMTR2, CST7, AKNA, RIBC2, IFI27, SPG11, KRTAP2-3, GLI3, POMC, CDO1, PCYOX1L, PLEK2, CCN2, TGFBI, BLMH, GNG11, IGFBP1, NLRP2, LDB2, GPX1, NUP85, ZDHHC7, RAB34, TPBG, SERPINB4, EGR1, RNF128, AXL, SRPX, TRIB2, NTN4, ZNF100, H1FX, CDH1, IGFBP2, EPS8L3, RNFT2, LXN, PHGDH, MEST, CPPED1, ALDH1A3, LIMA1, SELENOW, EDNRB, NRIP1, GCH1, FOXC1, CDKN1C, GRAMD2B, ANXA3, IFIT1, PLPP3, OSBPL3, CAV1, LAMA3, RPTN, RFX8, CRYBG2, C19orf33, EBP, MAP2K1, ART3, IFI27L2, AGAP3, CYTL1, SIPA1, IL37, GATA6, FGFBP2, ADRB2, ST3GAL5, PABPC4L, A2M, NDFIP2, REXO5, SLC30A1, METTL22, ARSG, CLK1, MMP14, CSRP1, METTL7B, OCIAD2, TSPAN6, PTGS1, RARRES1, ETV5, NES, RFTN1, SPP1, CALML3, TSPAN33, DBN1, SSBP3, CPQ, SHD, ERBIN, KLK1, HSPG2, LIFR, DUSP2, SMYD4, RINL, ZNF239, MAP3K5, KRT23, FXYD3, ITGA2, CCN3, TSPO, IVNS1ABP, HRH1, DUSP10, DUSP23, IER5, CEBPD, ATP6V0A4, C6orf226, TOR4A, LTBP2, LMO3, COCH, ERI1, DKK1, PPP1R26, IGFBP4, TYRP1, NRCAM, TRIM51, RAB20, ROR2, GPD2, PEG10, GALNT5, POGLUT3, KIF5B, VGF, IFI30, SKAP2, PTPRM, ARHGAP18, GAPDHS, FAT1, PIM1, CST6, MPO, CXCR4, EPB41L3, DYNLT3, CXCL8, PSCA, CYP1B1, CXCL3, GLB1L, DUSP5, BLNK, SSBP2, NLGN4X, PAQR8, ASPH, CLEC2L, KCNN4, PAEP, UPP1, RBM20, NDRG1, PRSS1, SHROOM2, MED12, KANK1, CDCA7, PCLAF, EVA1C, FAP, TYMSOS, CKB, PHLDA1, SIRPA, CASP4, CYP3A5, TLR7, CAMK2N1, HLX, ENO2, FSD1, HYI, GSDME, TNFSF10, NRN1, ZNF91, TMEM141, ZSCAN16, ABHD12, F3, ITM2A, GASK1B, THSD7A, RBMS1, BEX1, GLCE, SHC1, ITGB1BP1, IFITM2, COLEC12, GPR183, GEM, SFRP1, ZNF544, PTGFRN, CALML5, DRAM1, MET, PCP4, GMPR, SMCO4, CLEC11A, MLPH, PROS1, SEMA5A, WDR54, LPAR6, GBP2, SNCAIP, GNG12, SGCD, KCNK12, INHBE, GCNT3, RHOH, NADSYN1, EPAS1, C15orf48, ZNF268, ZNF92, MAGEH1, FRMD6, TBL1X, TM4SF18, MYCN, VOPP1, CA2, PTGER2, SERPINA1, SCCPDH, CLEC2B, MFGE8, GPRC5A, PARD6G, TUBA3C, TEX19, ALDH2, GSN, FUCA1, SUMF2, EPHA2, METTL7A, HTATIP2, TBC1D2, PRDX2, ATM, ZBED3, TPST1, PHGR1, DDX3Y, NELL1, PECR, CD44, GPX7, HLTF, TMEM167A, MAP1B, ITGA3, DDIT4 , CHGB, ZFPM1, PLSCR1, SNAI2, DOCK11, ZNF131* |

| Predictor genes list for drug Nilotinib |
| --- |
| *GPNMB, PHGDH, TACSTD2, GPR65, PROK2, CPPED1, ALDH1A3, BEX3, GAREM1, LIMA1, ENPEP, GDF15, BCAS3, UBASH3B, SNX19, ANK1, C1QL1, SPATS2L, PARP8, NXF3, SNX7, LAYN, HLA-C, BASP1, FOXC1, S100A14, CPED1, SIX4, RP11-388M20.9, S100A2, CSMD1, IGLL1, PERP, CAV1, C1QTNF4, TM4SF1, PRSS23, GADD45B, EMP1, MS4A3, RAB27A, STC1, RAB33A, CCDC173, GNG12, SPA17, TUBAL3, 43891, FERMT3, ANTXR2, C19orf33, KIF21A, MTO1, BIN1, FADS1, SCML1, MGST3, CA8, RASGEF1A, ACBD3, SDC4, ZDHHC14, FUT7, PLIN2, FOS, RHOH, PIM1, FAT1, APP, C1orf186, BST2, RASGRP2, FAM162B, AEBP1, C15orf48, ENC1, ARNTL2, WASF1, TDRD9, TSPAN13, PABPC4L, CST7, TSC22D3, EMP2, SGCG, BTG3, TRPC1, GPC4, STAT4, TBXA2R, CNN3, C3orf14, TMEM154, BBS12, GPX8, LCP1, KCNN4, CLCA1, ZBED8, SAMSN1/LOC388813, ALOX5AP, PIR, DUSP1, CLEC2B, NKD2, S100A13, FZD6, CLDN1, TESC, SEC31B, KAT2B, EN2, PPDPF, TRIM13, LGMN, SPRR2D, LPXN, PCDHB5, FAR2, MLF1, GTSF1, SELL, ITPRIPL2, ST6GALNAC2, SLAIN1, FUCA1, CISH, SOCS2, CYBA, ANKRD27, MGLL, ARHGEF5, PYCARD, MAGED2, ADM, CSRP2, C20orf197, ATM, CD52, GAS6, TMTC1, DENND3, B3GNT2, COL15A1, VIM, PSTPIP2, TRIM22, HYI, MFHAS1, S100A10, SCG2, PKN1, OSCAR, CTSF, ARHGAP10, DOCK2, C9orf64, CGAS, RGS10, ICAM3, PRSS57, SQOR, AXL, ZBTB16, LOC339803, ANKRD55, DNAJC12, CFAP161, MOXD1, CBR3, RTL8A, NGEF, LITAF, KRT7, HMGN5, HTRA1, S100A4, SELENOP, FAM71E1, FHIT, CAMK2D, SOX9, PLK2, TPD52L1* |

| Predictor genes list for drug Nutlin-3a |
| --- |
| *MSN, FNBP1, MEST, IFITM2, SOX21, DDR2, TGM1, GPR21/RABGAP1, APOBEC3G, ACKR4, IGFN1, SFRP1, NAPRT, DSTN, COL3A1, GDF15, HIPK2, DRAM1, SPINK1, NFIB, CXCL2, C8orf31, VWDE, FOXA1, EDNRB, RFLNB, RPS27L, LAMB3, ETS2, DLG1, TJP2, GRAMD2B, CMTM3, AASS, TMEM100, CDKN2A, PERP, PTPRD, TRIM51, LGALS1, ZNF608, XYLT1, BOLA1, ISG15, FKBP14, ST6GALNAC5, KCNK1, USP43, JAG2, CTNNAL1, CDK6, ST14, C19orf33, GSTO1, ASS1, CRADD, SCML1, DDX1, CA9, VAMP8, FES, FBXL16, KPTN, MEF2C, LTBR, TPK1, CPD, GAL, DLG3, PHLDA3, BST2, KLHL36, FDXR, C15orf48, BEX5, RUFY3, SERPINB8, PAGE4, MMP8, DENND5A, AGR3, HPD, IFNLR1, FKBP4, AKR1C1, ACAT2, AIM2, ZNF600, BTG3, CT83, INSM1, STAG2, HRCT1, BAX, MIB2, ENPP2, ZDHHC23, PKP2, GPSM2, SERPINA1, KCNS3, MYBPH, FOXG1, GLDC, CD82, SNX9, SAMSN1/LOC388813, BBC3, CCL28, PLEKHA5, SLC27A2, POU4F1, COLEC10, PGD, MICALL2, VAMP5, SUPT20H, OCIAD2, ANK3, GPR87, CLGN, NAV3, KRT8, GPRC5A, GYPC, XPC, RPL22L1, C2CD4A, TXNIP, LRRC17, CKB, BICD1, TSPAN8, SELL, ST6GALNAC2, LAPTM4B, ADAMTS9, NUP62CL, DSC2, KCNMA1, ALPK2, SPP1, DSP, PLK3, CHKA, CAMK2N1, MGST2, GSTT1, RERG, DDX3Y, TIPARP, PAG1, KRT19, EGR1, PTGS2, SLC47A2, SPARC, TRIM22, HLTF, DSG2, CSTF2T, SH3BGRL, CXXC4, RINL, RNF128, MORN2, SMOC1, RNPS1P1, TNFSF10, CTDSPL, WDFY2, NRTN, ZNF423, BOD1L1, SRGN, ZSWIM7, ADSSL1, NMU, LOC339803, CTHRC1, SYT17, ONECUT2, PRSS21, RASL10B, TMEM56, LAPTM5, KRT7, MDM2, HLA-DRA, GRB7, MSANTD4, FHIT, SYCP2, SLC26A2, LRMP, STXBP5, CORO1A, UBE2E2* |

| Predictor genes list for drug PD-0325901 |
| --- |
| *SLC22A4, ANXA1, MMP28, GALNT1, CD302, TNFRSF11B, MMP1, KRT13, BASP1, KANK4, LRRK2, CTHRC1, ZFPM2, HMGCS1, C1orf116, GLMP, TSPAN6, GSAP, ERRFI1, GLCE, HMGN5, MAGEF1, BEX4, KANK1, IGHM, RECQL4, SHD, SOWAHC, SNX8, RAB32, HHEX, TAFA5, ITPRIPL2, IGFBP3, IRX1, LDLR, LCN2, PSG4, TUBB2B, ID4, DOCK11, TUBA3C, HTRA1, ZNF738, COQ10A, GALNT3, OCIAD2, SERPINB1, SLC36A4, ONECUT2, CCN2, CST1, STAC3, ICAM1, SLC38A1, TESPA1, ABCG1, PMP22, PTGER4, ITGA2, POPDC3, RERG, RAB20, GJB2, MT1E, POU4F1, TSPO, MSN, CMTM3, ELOVL2, EYA4, FUCA2, CCDC74A, MITF, CRYBG2, FGB, DSCAML1, METTL22, PCDHB5, COLGALT2, MGST1, NFKBIZ, AC241585.1, ABCC3, ENC1, NNMT, VSIG1, SPP1, KCNMB4, LGALS8, DSG2, COLEC12, OLIG2, TMEM144, TBC1D2, CTNNA1, TOM1L1, SRPX, SH3BP5, ARRDC2, GUCY1B1, FCER1G, IER3, BLNK, CLEC11A, THBS2, PDZK1IP1, NPTN, NRIP1, IGFBP2, OGFOD3, HLA-DRA, LIMA1, SH3PXD2B, COL18A1, APOBEC3B, IL24, VEGFC, PMEPA1, GMPR, CBR1, STC2, ENO2, SCN2A, LYZ, CFI, ST3GAL6, RELN, MMD, SPRY2, TSPAN1, TRIB2, PABPC4L, GPD1L, ARHGAP18, IGFBP5, LGMN, HSPA2, GDA, CA8, TIMP3, VEPH1, NRN1, SLC1A4, ANXA2, CALB1, ZNF22, NEDD9, HOXB2, LGALS1, CACNA2D4, HENMT1, TDRD9, GYPC, UCA1, PTPRZ1, MYEF2, DBP, C15orf48, CXCL8, VGF, SLPI, DUSP23, CXCL3, TUBA4A, CENPV, CP, PTGS2, PXDN, S100B, MYO1D, IGFBP1, GDF15, TRABD2A, SPIRE1, ASPH, PDK4, TMEM101, COCH, VPS37B, NQO2, IFITM2, AC026412.1, ZFP36, COL4A5, LUM, PPARG, PLPP1, MGARP, ALKBH3, RAD51AP1, SLC44A2, KCNH2, CDKN2A, TIAM2, EMP1, CYBRD1, GSTT1, ITM2A, NR2F2, PCCA, SLC44A1, FAM229B, TYRP1, CELSR1, CMTM8, PGD, SLC22A15, AKR1B10, SUCNR1, PTGR1, GFRA1, CAMK2N1, PRDX2, LAMA4, SAMD5, SLC7A5, PHACTR2, SHFL, PSMB8, TMSB15B, PCP4, HLA-DRB1, NDFIP2, DCT, GSTO2, SMIM14, SYTL3, SLC26A2, CYSTM1, STEAP1B/LOC401312, S100P, CAT, SH3BGR* |

| Predictor genes list for drug PD-0332991 |
| --- |
| *GPR19, FARP1, SKA2, LPAR6, S100A13, MMP1, ADCY7, GZMA, CLDN4, MLF1, FNDC1, SYCP2L, GSDMB, TWIST1, SERPINF1, CD248, RASGEF1A, ID1, MPP1, SOWAHC, TFPI, ID4, MAPKAPK3, BAALC, LINC00839, BIN1, ECSCR, TTC27, RP11-1055B8.7, CD44, CRNDE/LOC101927480, NEFH, CTNNA2, PROX1, UBE2L6, KRT23, PRXL2A, C1orf174, NLRC3, AKAP12, LIFR, STAT5A, REC8, FBXL13, NEXN, MESP1, GPD2, PPP1R3B, PKIB, PSAT1, SNU13, SOCS2, HPS3, CADM1, CASP4, WASHC3, RBP7, AC093840.1, IGFLR1, DHRS2, CDH4, MGST1, DIAPH2, CDX2, TMEM178A, MND1, PDGFRA, CBFA2T3, ERAP2, ACAT2, LMAN1, SNX30, BACH2, FAM111B, RAP2A, PAPPA, CABYR, FAIM, CSTF2T, AGT, C1orf54, PDK3, KRT7, CLDN10, FBXL16, COLCA2, VWDE, AMPH, HTR3A, GDPD5, NLRP2, TMEM97, WASF3, C4orf19, NUP62CL, RORA, CRADD, NUDT19, PLS1, COL23A1, CALML5, LACTB2, IGFBP6, NR2F1, C2CD4B, C9orf64, RPL27A, USP44, MOB3B, SHLD1, TMEM56, GYPC, INTS9, ASRGL1, FBXO33, PCNX4, ERBB3, SLC16A14, C3orf14, GATM, CAV1, GNA11, TUBA4A, HBEGF, ABCA1, TOP1MT, EMC9, RASGRP2, ZNF773, BMP7, KRT8, STXBP6, SELL, TPD52L1, CDH13, KCNS3, CACNA2D3, TRIM66, SLFN11, IFITM2, SIPA1L2, LPXN, RIPPLY3, PIP5K1B, IL23A, FAM43A, IFI16, PYCARD, ARL4C, SCRN2, PTPRE, RB1, PLPP1, GPX2, PHACTR3, CD38, ARHGEF26, KIAA1671, SLC2A10, TNKS, ATP1B1, ERICH2, LYRM1, CDKN2A, GSTO1, CDKAL1, SAA1, HSD3B1, MOSPD1, ZNF469, SELENOP, MPST, LONRF1, CDC42BPA, PYROXD2, SYBU, PTPRC, LMO2, HERC5, PLPP2, TOX3, SLC7A5, ARMH1, KCNK1, NPY, PCP4, PRKCB, RTN1, QPCT, S100A14, HOXD13, CXADR, CCNE1, CYB5A, TUBB2B* |

| Predictor genes list for drug PHA-665752 |
| --- |
| *IFNLR1, RXFP2, ARHGAP24, CCDC91, CCN1, SLC25A37, PTGDS, SETD7, FAAH2, CDKN2A, SDC1, MEST, CYBRD1, METTL7B, YWHAG, NR2F2, SLN, LINC02308, S100A4, ENTPD3, MFNG, STK26, HTR1F, NTSR1, CLMP, CAPN2, PDE7A, PTPRC, PTGR1, LDHB, GMFG, RECK, NQO1, GNG12, CHRNA9, SPON2, NMUR2, NR5A2, VWA5A, TPD52L1, WDR60, LAPTM5, PTPRH, ARHGAP15, SPATS2L, VAMP5, COA1, ADGRA2* |

| Predictor genes list for drug Erlotinib |
| --- |
| *FST, S100A16, PTAFR, ZNF667-AS1, TCF4, HSPA2, PPBP, BMP7, ATP6AP1L, EGR1, RGCC, INPP1, SERPINA1, S100A10, FERMT1, TM4SF4, BHMT, CLIC3, DCAF12L1, GPM6A, HOXA9, EFEMP1, CXCL1, NMB, DKK1, IFITM3, MMP13, TGFBI, PDGFA, ALDH1A1, S100A7, ALDH3A1, KHDRBS3, DSC2, CAV1, KLK5, ARID5B, NRIP3, XYLT1, PI3, ITGB6, PPP1R14C, PLK2* |

| Predictor genes list for drug AZD-0530 |
| --- |
| *HK2, IGFBP3, VCAM1, MUC16, MXRA5, PTHLH, PLEKHA4, DERL3, ZNF439, LUM, ST3GAL6, DKK1, ZNF69, ANXA3, MID1IP1, DCN, S100A2, TGFB1, RARRES2, DLL3, HPSE, SRPX2, PIGR, C19orf33, CDH6, PLGRKT, VGF, CLIC3, CREBL2, ALX1, PPIE, BST2, NFKBIZ, COL1A2, SLC16A6, PDGFC, MANEAL, VEGFC, AKR1C2, INSM1, TMEM216, ARRDC2, GBP1, CCL28, CAST, EPYC, BAG2, TGFBI, COPZ2, CCR6, CISH, ADM, TPM4, CTSH, SIGLEC6, RAB34, TPM2, PDLIM1, CXXC5, TFPI, ARL9, PSTPIP2, RAB11FIP1, OLFML2A, AHR, MYOF, SCGB1A1, CD81, C1R, KRT4, OTUD1, RRAGD, DDIT4, AAMDC, TIAM2, HOMER3, PKIB, TMEM170B, RBMS1, GPAT3* |

| Predictor genes list for drug PLX4720 |
| --- |
| *HIST1H2BD, CYP27A1, BASP1, KIAA1549L, ACSS1, PROS1, LEF1, PDGFC, CNIH3, TNFSF10, ZNF239, SERPINF1, TMPRSS3, RASGEF1A, DLL3, ID1, SLC25A4, SH3BGRL2, MKLN1, DSP, SERPIND1, CYTL1, HLA-DPA1, SPON2, BCAS3, RORB, S1PR3/C9orf47, ETV1, TBX2, PREX1, ONECUT2, VAMP8, SOX8, KIAA1324L, KRT23, NSG1, CD109, LIFR, LHX8, REC8, CSRP2, ACTR3C, TACSTD2, SLC16A4, GLYATL2, ENPP2, SEPTIN2, ELOVL2, MMP8, EYA4, BST1, GADD45B, MGAM, SGK1, CRYBG2, IL17RB, KIF26A, CEACAM6, CDX2, AK5, TMEM163, MANF, SFRP1, PPARGC1A, SPRR2D, TF, ZNF862, CFD, TWSG1, FXYD3, GIMAP2, GUCY1B1, MTSS1, SNAI2, BTN3A2, CLEC11A, FGFR3, PPP2R2C, THBS2, PDK3, IL24, GAPDHS, VAMP5, GPRC5C, THY1, STC2, GSE1, MPPED2, RAB42, MAL2, NUP62CL, ST3GAL6, PLS1, A1BG, SIRPA, TIMP3, SDC1, VIM, CEP70, BCL2A1, VLDLR, ELAVL2, LGALS1, GRHL3, WIPI1, CCDC50, HOXA7, TRAF3IP2, PLBD1, ADRA2C, VGF, CXCL1, GATM, PLA2G4A, PXDN, FBP1, HAS2, LRRC75B, ALKBH4, FAH, NES, SNCA, KRT8, SYNGR3, UCHL1, CCSER2, APOC1, ARHGAP23, PRSS33, GPR37, CD200, DDX59, KRT19, APOD, SETD6, NCALD, TM4SF18, 43891, CD14, APOE, SCGN, ST6GAL1, RFLNB, SERP2, TM4SF19, CCDC3, ABLIM1, NEURL1B, PUDP, IRAK1, LAMA4, WFDC1, SAMD11, GSTP1, MEGF10, GPR137B, FKBP1A, SPINT2, LOXL1, COL5A1, QPCT, DCT, ID2, GSTO2, HOXD13, SLC26A2, SNTB1, STEAP1B/LOC401312, THSD7A, BCHE, LLGL2* |

| Predictor genes list for drug Sorafenib |
| --- |
| *TGFBR1, IQGAP2, P4HA1, SRGN, NPM3, CHGA, DNMT3B, ESRP1, WFDC2, EIF4EBP1, SGCB, SIX4, TSPAN6, IGLL1, EEF1A1, GLCE, TNFSF10, FERMT3, CD1D, NMNAT2, THBD, PXK, RGS17, CELF2, BEX4, CLEC2B, CPT1A, PTPRR, UBAC2, PARM1, CA2, MPP1, HHEX,* *LOC728138, IGLL5, NKD2, DOCK11, MAPKAPK3, JARID2, OCIAD2, SERPINB1, RP11-1055B8.7, ANTXR2, MARCKS, SH2D3C, MAGI3, HLX, TSPAN13, ARHGAP5, CCDC102A, ITGB2, SCG2, SPATS2L, HCCS, GSTM3, S100A9, PRXL2A, ICA1, CD93, TARBP1, POPDC3, NBEA, SBK1, REC8, POU4F1, NDNF, ATP8B4, MESP1, BAG2, PIWIL4, ZNF431, CD164, FAM177A1/LOC101927178, PIEZO1, SOCS2, HDGFL3, CMBL, NEUROG2, IL17RB, RGS18, PPP1R26, IRF8, GSTA4, CALCB, PRTFDC1, MYC, RTL8A, ALX1, KLF12, FHDC1, NLGN4X, GIMAP2, TYROBP, LAT2, CHST11, CD300A, CCL5, FAIM, MPO, ISL1, C1orf54, WT1, FAM45A, FBXL16, MRGPRF, ICAM2, DNER, STAR, BHLHE22, PLEK, LYZ, AP001007.1, NCF4, CFP, CAPRIN2, ZNF521, AIF1, RORA, CEACAM21, MICALCL, ZDHHC2, SMARCA1, AMN1, INA, SOWAHD, DLX6, METTL7B, XBP1, MATK, CHN1, ANK3, NRG4, LRMP, RNASEH2B, DSTN, LGALS1, CYB5A, CCDC50, PTPRK, GLB1L2, GJC1, EFNA5, MYEF2, STK10, GABRB3, VAV1, POLR3D, AFAP1, MCOLN3, CPPED1, LGALS12, TPST1, ZNF711, ZBTB20, C1orf162, RUNX1T1, EMILIN2, LURAP1L, DHRS4, CTPS2, SNTB2, SNAP25, GALNT18, MANEAL, IRAK1BP1, BCAT1, LRRC75A, JAG2, GEM, SLFN11, AC026412.1, NFIL3, UCHL1, PROK2, HOXC4, RIMBP2, GANC, ST3GAL1, TFAP2E, CRIPAK, JUN, BMI1, ZNF518B, GBE1, RNASE2, SYK, EMILIN1, ZNF502, ZNF415, KCNN4, DYNLT3, ATP1B1, TRIO, NDFIP1, CNN3, TUSC3, SYT1, OAF, ANGPT1, S100A4, ERICH5, HTRA3, CAST, XYLT1, SLC6A15, PIR, TMEM14A, ARHGDIB, LMO2, AOAH, BID, SUCNR1, ITGAL, TRIM32, PCLO, CKAP4, PRDX2, ADA, PNMA8A, IRAK3, PLXND1, OCSTAMP, TOX3, HOXA11, UBA7, CD68, SHCBP1, PSTPIP2, FXYD5, BST2, EVI2A, N4BP2L1, NMI, TFEC, KHDRBS3, DTD1, ATG16L2, TP53RK, TCEAL9, ARHGAP15, HBB, HINT2, TUBB2B, HCK* |
