# Supplementary material for: Machine learning based anti-cancer drug response prediction and search for predictor genes using cancer cell line gene expression
Source: Genomics Inform. 2021 Mar 26;19(1):e10. doi: 10.5808/gi.20076 (PMC8042299; doi:10.5808/gi.20076)
Supplement: Supplemental Table 2. — Gene ontology analysis results on the predictor genes for the 12 drugs in GDSC. We used Metascape to perform gene enrichment analysis to determine the gene functions, related pathways and their enrichment levels of common predictor genes we found for 12 drugs. (The number of common predictor genes for each drug is shown in Table 2) [file gi-20076suppl5.docx]

**Supplemental Table 2** Gene ontology analysis results on the predictor genes for the 12 drugs in GDSC. We used Metascape to perform gene enrichment analysis to determine the gene functions, related pathways and their enrichment levels of common predictor genes we found for 12 drugs. (The number of common predictor genes for each drug is shown in Table 2).

**17-AAG**

| GO | Description | Count | % | Log10(P) | Log10(q) |
| --- | --- | --- | --- | --- | --- |
| GO:0043062 | extracellular structure organization | 25 | 9.62 | -11.81 | -7.46 |
| GO:0040017 | positive regulation of locomotion | 24 | 9.23 | -8.32 | -4.67 |
| GO:0070848 | response to growth factor | 26 | 10.00 | -8.01 | -4.51 |
| GO:0030155 | regulation of cell adhesion | 25 | 9.62 | -7.51 | -4.17 |
| GO:0043408 | regulation of MAPK cascade | 25 | 9.62 | -7.29 | -3.98 |
| GO:0006935 | chemotaxis | 23 | 8.85 | -7.22 | -3.97 |
| R-HSA-3000171 | Non-integrin membrane-ECM interactions | 8 | 3.08 | -7.14 | -3.97 |
| GO:0008285 | negative regulation of cell proliferation | 25 | 9.62 | -6.97 | -3.85 |
| GO:0048608 | reproductive structure development | 18 | 6.92 | -6.67 | -3.60 |
| GO:0097435 | supramolecular fiber organization | 23 | 8.85 | -6.51 | -3.51 |
| GO:0031960 | response to corticosteroid | 11 | 4.23 | -6.38 | -3.41 |
| R-HSA-198933 | Immunoregulatory interactions between a Lymphoid and a non-Lymphoid cell | 10 | 3.85 | -6.33 | -3.40 |
| GO:0042060 | wound healing | 20 | 7.69 | -6.08 | -3.18 |
| GO:0002263 | cell activation involved in immune response | 22 | 8.46 | -5.90 | -3.07 |
| R-HSA-109582 | Hemostasis | 20 | 7.69 | -5.77 | -2.99 |
| GO:0010035 | response to inorganic substance | 19 | 7.31 | -5.75 | -2.98 |
| GO:0001503 | ossification | 16 | 6.15 | -5.73 | -2.98 |
| GO:0051347 | positive regulation of transferase activity | 21 | 8.08 | -5.59 | -2.90 |
| R-HSA-381426 | Regulation of Insulin-like Growth Factor (IGF) transport and uptake by Insulin-like Growth Factor Binding Proteins (IGFBPs) | 9 | 3.46 | -5.57 | -2.89 |
| GO:0050769 | positive regulation of neurogenesis | 17 | 6.54 | -5.47 | -2.85 |

**AZD-0530**

| GO | Description | Count | % | Log10(P) | Log10(q) |
| --- | --- | --- | --- | --- | --- |
| GO:0001667 | ameboidal-type cell migration | 10 | 12.50 | -5.92 | -1.86 |
| M5884 | NABA CORE MATRISOME | 8 | 10.00 | -5.91 | -1.86 |
| GO:0070848 | response to growth factor | 11 | 13.75 | -5.04 | -1.48 |
| GO:0001501 | skeletal system development | 9 | 11.25 | -4.77 | -1.38 |
| GO:0006027 | glycosaminoglycan catabolic process | 4 | 5.00 | -4.55 | -1.32 |
| GO:0019216 | regulation of lipid metabolic process | 8 | 10.00 | -4.49 | -1.32 |
| GO:0002366 | leukocyte activation involved in immune response | 10 | 12.50 | -4.42 | -1.32 |
| M18 | PID INTEGRIN1 PATHWAY | 4 | 5.00 | -4.41 | -1.32 |
| GO:0001101 | response to acid chemical | 7 | 8.75 | -4.21 | -1.24 |
| GO:0070613 | regulation of protein processing | 5 | 6.25 | -3.75 | -0.97 |
| GO:0001503 | ossification | 7 | 8.75 | -3.74 | -0.97 |
| GO:0001933 | negative regulation of protein phosphorylation | 7 | 8.75 | -3.55 | -0.85 |
| GO:0019932 | second-messenger-mediated signaling | 7 | 8.75 | -3.48 | -0.84 |
| ko04672 | Intestinal immune network for IgA production | 3 | 3.75 | -3.42 | -0.83 |
| GO:0048545 | response to steroid hormone | 6 | 7.50 | -3.31 | -0.75 |
| GO:0016331 | morphogenesis of embryonic epithelium | 4 | 5.00 | -3.03 | -0.58 |
| GO:0007162 | negative regulation of cell adhesion | 5 | 6.25 | -2.76 | -0.41 |
| ko05410 | Hypertrophic cardiomyopathy (HCM) | 3 | 3.75 | -2.76 | -0.41 |
| GO:1901654 | response to ketone | 4 | 5.00 | -2.58 | -0.30 |
| GO:0032006 | regulation of TOR signaling | 3 | 3.75 | -2.50 | -0.27 |

**AZD6244**

| GO | Description | Count | % | Log10(P) | Log10(q) |
| --- | --- | --- | --- | --- | --- |
| GO:0009611 | response to wounding | 33 | 10.31 | -10.99 | -6.65 |
| GO:0030155 | regulation of cell adhesion | 32 | 10.00 | -9.87 | -5.83 |
| GO:1902532 | negative regulation of intracellular signal transduction | 27 | 8.44 | -9.04 | -5.32 |
| GO:0043408 | regulation of MAPK cascade | 31 | 9.69 | -8.96 | -5.32 |
| GO:0008285 | negative regulation of cell proliferation | 31 | 9.69 | -8.56 | -5.00 |
| GO:0001568 | blood vessel development | 31 | 9.69 | -8.49 | -4.99 |
| GO:0048732 | gland development | 22 | 6.88 | -7.96 | -4.56 |
| GO:0031099 | regeneration | 15 | 4.69 | -7.90 | -4.56 |
| GO:0048589 | developmental growth | 27 | 8.44 | -7.73 | -4.43 |
| M3008 | NABA ECM GLYCOPROTEINS | 14 | 4.38 | -7.18 | -4.01 |
| GO:0043062 | extracellular structure organization | 21 | 6.56 | -7.05 | -3.91 |
| GO:0052547 | regulation of peptidase activity | 21 | 6.56 | -6.88 | -3.76 |
| GO:0090066 | regulation of anatomical structure size | 22 | 6.88 | -6.74 | -3.71 |
| GO:0097237 | cellular response to toxic substance | 15 | 4.69 | -6.65 | -3.67 |
| GO:0001704 | formation of primary germ layer | 11 | 3.44 | -6.60 | -3.63 |
| GO:0001503 | ossification | 19 | 5.94 | -6.45 | -3.52 |
| M145 | PID P53 DOWNSTREAM PATHWAY | 11 | 3.44 | -6.27 | -3.37 |
| GO:0000904 | cell morphogenesis involved in differentiation | 26 | 8.12 | -6.17 | -3.29 |
| GO:0009617 | response to bacterium | 26 | 8.12 | -6.16 | -3.29 |
| GO:0007420 | brain development | 26 | 8.12 | -6.08 | -3.26 |

**Erlotinib**

| GO | Description | Count | % | Log10(P) | Log10(q) |
| --- | --- | --- | --- | --- | --- |
| M5885 | NABA MATRISOME ASSOCIATED | 11 | 25.58 | -7.90 | -3.55 |
| GO:0043062 | extracellular structure organization | 9 | 20.93 | -7.57 | -3.53 |
| GO:0061448 | connective tissue development | 7 | 16.28 | -6.83 | -3.18 |
| GO:0043588 | skin development | 8 | 18.60 | -6.62 | -3.05 |
| GO:0072124 | regulation of glomerular mesangial cell proliferation | 3 | 6.98 | -6.26 | -2.80 |
| GO:0019730 | antimicrobial humoral response | 5 | 11.63 | -5.65 | -2.45 |
| GO:0001568 | blood vessel development | 8 | 18.60 | -4.61 | -1.71 |
| GO:0030155 | regulation of cell adhesion | 7 | 16.28 | -3.92 | -1.13 |
| GO:0042475 | odontogenesis of dentin-containing tooth | 3 | 6.98 | -3.43 | -0.81 |
| GO:0032970 | regulation of actin filament-based process | 5 | 11.63 | -3.42 | -0.80 |
| GO:0002576 | platelet degranulation | 3 | 6.98 | -2.99 | -0.56 |
| GO:0050663 | cytokine secretion | 3 | 6.98 | -2.96 | -0.56 |
| GO:0046718 | viral entry into host cell | 3 | 6.98 | -2.93 | -0.55 |
| R-HSA-913531 | Interferon Signaling | 3 | 6.98 | -2.45 | -0.26 |
| M3468 | NABA ECM REGULATORS | 3 | 6.98 | -2.23 | -0.14 |

**Lapatinib**

| GO | Description | Count | % | Log10(P) | Log10(q) |
| --- | --- | --- | --- | --- | --- |
| GO:0040017 | positive regulation of locomotion | 28 | 12.23 | -12.54 | -8.19 |
| GO:0043588 | skin development | 23 | 10.04 | -11.96 | -8.19 |
| M5885 | NABA MATRISOME ASSOCIATED | 29 | 12.66 | -11.31 | -7.67 |
| GO:0009611 | response to wounding | 28 | 12.23 | -11.18 | -7.61 |
| GO:0097191 | extrinsic apoptotic signaling pathway | 16 | 6.99 | -10.06 | -6.76 |
| GO:0052547 | regulation of peptidase activity | 21 | 9.17 | -9.44 | -6.21 |
| GO:0002274 | myeloid leukocyte activation | 24 | 10.48 | -8.85 | -5.71 |
| GO:0030155 | regulation of cell adhesion | 25 | 10.92 | -8.63 | -5.54 |
| GO:0045862 | positive regulation of proteolysis | 17 | 7.42 | -7.75 | -4.80 |
| GO:0032103 | positive regulation of response to external stimulus | 16 | 6.99 | -7.63 | -4.72 |
| GO:0009991 | response to extracellular stimulus | 20 | 8.73 | -7.52 | -4.62 |
| GO:0001525 | angiogenesis | 21 | 9.17 | -7.43 | -4.56 |
| GO:0008285 | negative regulation of cell proliferation | 24 | 10.48 | -7.42 | -4.56 |
| GO:0031344 | regulation of cell projection organization | 23 | 10.04 | -7.38 | -4.55 |
| GO:0033627 | cell adhesion mediated by integrin | 8 | 3.49 | -6.92 | -4.20 |
| GO:0032496 | response to lipopolysaccharide | 15 | 6.55 | -6.91 | -4.20 |
| GO:0030198 | extracellular matrix organization | 16 | 6.99 | -6.69 | -4.07 |
| GO:0010035 | response to inorganic substance | 19 | 8.30 | -6.59 | -3.99 |
| M18 | PID INTEGRIN1 PATHWAY | 7 | 3.06 | -5.94 | -3.40 |
| GO:0001666 | response to hypoxia | 14 | 6.11 | -5.75 | -3.24 |

**Nilotinib**

| GO | Description | Count | % | Log10(P) | Log10(q) |
| --- | --- | --- | --- | --- | --- |
| GO:0030036 | actin cytoskeleton organization | 20 | 10.99 | -7.37 | -3.25 |
| GO:0002683 | negative regulation of immune system process | 16 | 8.79 | -6.80 | -3.06 |
| R-HSA-109582 | Hemostasis | 17 | 9.34 | -6.15 | -2.50 |
| GO:0003014 | renal system process | 8 | 4.40 | -5.77 | -2.36 |
| GO:0002274 | myeloid leukocyte activation | 17 | 9.34 | -5.75 | -2.36 |
| GO:0001823 | mesonephros development | 7 | 3.85 | -5.29 | -2.06 |
| GO:0010631 | epithelial cell migration | 12 | 6.59 | -5.27 | -2.06 |
| GO:0051592 | response to calcium ion | 8 | 4.40 | -5.23 | -2.06 |
| GO:0050878 | regulation of body fluid levels | 14 | 7.69 | -5.11 | -2.02 |
| GO:0050900 | leukocyte migration | 14 | 7.69 | -5.09 | -2.02 |
| GO:0055074 | calcium ion homeostasis | 13 | 7.14 | -4.88 | -1.85 |
| GO:0040017 | positive regulation of locomotion | 15 | 8.24 | -4.84 | -1.83 |
| GO:0006907 | pinocytosis | 4 | 2.20 | -4.78 | -1.80 |
| GO:0031579 | membrane raft organization | 4 | 2.20 | -4.64 | -1.75 |
| GO:0010942 | positive regulation of cell death | 16 | 8.79 | -4.47 | -1.65 |
| GO:0040015 | negative regulation of multicellular organism growth | 3 | 1.65 | -4.37 | -1.61 |
| GO:0043122 | regulation of I-kappaB kinase/NF-kappaB signaling | 9 | 4.95 | -4.37 | -1.61 |
| GO:0051345 | positive regulation of hydrolase activity | 16 | 8.79 | -4.25 | -1.60 |
| GO:0032496 | response to lipopolysaccharide | 10 | 5.49 | -4.15 | -1.56 |
| GO:0097202 | activation of cysteine-type endopeptidase activity | 3 | 1.65 | -3.94 | -1.45 |

**Nutlin-3a**

| GO | Description | Count | % | Log10(P) | Log10(q) |
| --- | --- | --- | --- | --- | --- |
| M145 | PID P53 DOWNSTREAM PATHWAY | 12 | 6.09 | -9.57 | -5.22 |
| GO:0070268 | cornification | 10 | 5.08 | -8.11 | -4.06 |
| GO:0097435 | supramolecular fiber organization | 21 | 10.66 | -7.37 | -3.72 |
| GO:0072332 | intrinsic apoptotic signaling pathway by p53 class mediator | 8 | 4.06 | -7.10 | -3.61 |
| GO:0001503 | ossification | 15 | 7.61 | -6.57 | -3.31 |
| hsa01524 | Platinum drug resistance | 7 | 3.55 | -6.07 | -2.96 |
| GO:0061458 | reproductive system development | 14 | 7.11 | -5.41 | -2.48 |
| GO:0052548 | regulation of endopeptidase activity | 13 | 6.60 | -4.83 | -2.05 |
| GO:0071407 | cellular response to organic cyclic compound | 14 | 7.11 | -4.74 | -2.00 |
| GO:0031667 | response to nutrient levels | 14 | 7.11 | -4.73 | -2.00 |
| GO:0048729 | tissue morphogenesis | 16 | 8.12 | -4.65 | -1.97 |
| GO:1903829 | positive regulation of cellular protein localization | 11 | 5.58 | -4.52 | -1.88 |
| GO:0031589 | cell-substrate adhesion | 11 | 5.58 | -4.27 | -1.76 |
| GO:0007219 | Notch signaling pathway | 8 | 4.06 | -4.12 | -1.63 |
| GO:0031650 | regulation of heat generation | 3 | 1.52 | -4.03 | -1.60 |
| GO:0001771 | immunological synapse formation | 3 | 1.52 | -3.83 | -1.46 |
| GO:0008285 | negative regulation of cell proliferation | 16 | 8.12 | -3.81 | -1.44 |
| GO:0043269 | regulation of ion transport | 15 | 7.61 | -3.80 | -1.44 |
| GO:0010721 | negative regulation of cell development | 10 | 5.08 | -3.77 | -1.42 |
| GO:0048066 | developmental pigmentation | 4 | 2.03 | -3.51 | -1.24 |

**PD-0325901**

| GO | Description | Count | % | Log10(P) | Log10(q) |
| --- | --- | --- | --- | --- | --- |
| M5884 | NABA CORE MATRISOME | 17 | 7.30 | -9.74 | -5.67 |
| GO:0071396 | cellular response to lipid | 24 | 10.30 | -9.72 | -5.67 |
| GO:0009617 | response to bacterium | 24 | 10.30 | -7.64 | -4.07 |
| GO:0048608 | reproductive structure development | 17 | 7.30 | -6.65 | -3.32 |
| GO:0030198 | extracellular matrix organization | 16 | 6.87 | -6.59 | -3.32 |
| GO:0048545 | response to steroid hormone | 15 | 6.44 | -6.58 | -3.32 |
| GO:0032355 | response to estradiol | 10 | 4.29 | -6.50 | -3.32 |
| GO:0010035 | response to inorganic substance | 19 | 8.15 | -6.47 | -3.32 |
| GO:0031667 | response to nutrient levels | 18 | 7.73 | -6.43 | -3.31 |
| GO:0002366 | leukocyte activation involved in immune response | 21 | 9.01 | -6.14 | -3.07 |
| R-HSA-109582 | Hemostasis | 19 | 8.15 | -5.89 | -2.92 |
| GO:0098754 | detoxification | 9 | 3.86 | -5.74 | -2.79 |
| GO:0045596 | negative regulation of cell differentiation | 21 | 9.01 | -5.64 | -2.71 |
| GO:0044706 | multi-multicellular organism process | 11 | 4.72 | -5.47 | -2.56 |
| GO:0043903 | regulation of symbiosis, encompassing mutualism through parasitism | 11 | 4.72 | -5.35 | -2.45 |
| M5885 | NABA MATRISOME ASSOCIATED | 20 | 8.58 | -5.26 | -2.41 |
| GO:0007613 | memory | 8 | 3.43 | -5.09 | -2.27 |
| GO:0009611 | response to wounding | 19 | 8.15 | -5.07 | -2.27 |
| GO:0071402 | cellular response to lipoprotein particle stimulus | 5 | 2.15 | -4.93 | -2.22 |
| GO:0002521 | leukocyte differentiation | 16 | 6.87 | -4.88 | -2.18 |

**PD-0332991**

| GO | Description | Count | % | Log10(P) | Log10(q) |
| --- | --- | --- | --- | --- | --- |
| ko04666 | Fc gamma R-mediated phagocytosis | 8 | 4.10 | -6.60 | -2.26 |
| hsa05200 | Pathways in cancer | 17 | 8.72 | -6.19 | -2.22 |
| GO:0071900 | regulation of protein serine/threonine kinase activity | 15 | 7.69 | -5.27 | -1.75 |
| R-HSA-202733 | Cell surface interactions at the vascular wall | 8 | 4.10 | -5.25 | -1.75 |
| GO:0051272 | positive regulation of cellular component movement | 16 | 8.21 | -5.18 | -1.75 |
| hsa04925 | aldosterone synthesis and secretion | 7 | 3.59 | -5.12 | -1.75 |
| GO:0045664 | regulation of neuron differentiation | 16 | 8.21 | -4.65 | -1.42 |
| GO:0008015 | blood circulation | 14 | 7.18 | -4.48 | -1.36 |
| GO:0050728 | negative regulation of inflammatory response | 8 | 4.10 | -4.13 | -1.19 |
| R-HSA-421270 | Cell-cell junction organization | 5 | 2.56 | -4.07 | -1.19 |
| ko05214 | Glioma | 5 | 2.56 | -4.07 | -1.19 |
| GO:0001819 | positive regulation of cytokine production | 12 | 6.15 | -3.97 | -1.17 |
| GO:0097190 | apoptotic signaling pathway | 14 | 7.18 | -3.97 | -1.17 |
| GO:0052547 | regulation of peptidase activity | 12 | 6.15 | -3.92 | -1.16 |
| GO:0055074 | calcium ion homeostasis | 12 | 6.15 | -3.92 | -1.16 |
| GO:0035051 | cardiocyte differentiation | 7 | 3.59 | -3.88 | -1.14 |
| GO:0030036 | actin cytoskeleton organization | 15 | 7.69 | -3.85 | -1.11 |
| GO:0051101 | regulation of DNA binding | 6 | 3.08 | -3.62 | -1.01 |
| GO:0014066 | regulation of phosphatidylinositol 3-kinase signaling | 6 | 3.08 | -3.57 | -0.98 |
| GO:0060973 | cell migration involved in heart development | 3 | 1.54 | -3.53 | -0.96 |

**PHA-665752**

| GO | Description | Count | % | Log10(P) | Log10(q) |
| --- | --- | --- | --- | --- | --- |
| GO:0006469 | negative regulation of protein kinase activity | 6 | 12.77 | -5.46 | -1.25 |
| ko04080 | Neuroactive ligand-receptor interaction | 5 | 10.64 | -4.00 | -0.74 |
| GO:0042542 | response to hydrogen peroxide | 4 | 8.51 | -3.97 | -0.74 |
| R-HSA-2142753 | Arachidonic acid metabolism | 3 | 6.38 | -3.87 | -0.67 |
| GO:0009792 | embryo development ending in birth or egg hatching | 6 | 12.77 | -3.08 | -0.06 |
| GO:0019932 | second-messenger-mediated signaling | 5 | 10.64 | -3.01 | -0.00 |
| GO:1905114 | cell surface receptor signaling pathway involved in cell-cell signaling | 5 | 10.64 | -2.41 | 0.00 |
| M3008 | NABA ECM GLYCOPROTEINS | 3 | 6.38 | -2.36 | 0.00 |
| R-HSA-418594 | G alpha (i) signalling events | 4 | 8.51 | -2.32 | 0.00 |
| GO:0051607 | defense response to virus | 3 | 6.38 | -2.02 | 0.00 |
| GO:0046434 | organophosphate catabolic process | 3 | 6.38 | -2.01 | 0.00 |

**PLX4720**

| GO | Description | Count | % | Log10(P) | Log10(q) |
| --- | --- | --- | --- | --- | --- |
|  |  |  |  |  |  |
| GO:0030155 | regulation of cell adhesion | 21 | 12.28 | -8.26 | -4.09 |
| GO:0030036 | actin cytoskeleton organization | 20 | 11.70 | -7.83 | -4.09 |
| GO:0048608 | reproductive structure development | 16 | 9.36 | -7.79 | -4.09 |
| GO:0045055 | regulated exocytosis | 20 | 11.70 | -6.99 | -3.54 |
| GO:0045596 | negative regulation of cell differentiation | 18 | 10.53 | -5.88 | -2.58 |
| GO:2000146 | negative regulation of cell motility | 12 | 7.02 | -5.56 | -2.32 |
| GO:0071396 | cellular response to lipid | 15 | 8.77 | -5.43 | -2.26 |
| GO:0061061 | muscle structure development | 16 | 9.36 | -5.31 | -2.16 |
| R-HSA-109582 | Hemostasis | 15 | 8.77 | -5.16 | -2.14 |
| GO:0001503 | ossification | 12 | 7.02 | -5.01 | -2.13 |
| GO:0050865 | regulation of cell activation | 15 | 8.77 | -4.90 | -2.10 |
| GO:0097191 | extrinsic apoptotic signaling pathway | 9 | 5.26 | -4.89 | -2.10 |
| GO:0045936 | negative regulation of phosphate metabolic process | 14 | 8.19 | -4.65 | -1.95 |
| GO:1901137 | carbohydrate derivative biosynthetic process | 16 | 9.36 | -4.61 | -1.94 |
| GO:0034447 | very-low-density lipoprotein particle clearance | 3 | 1.75 | -4.58 | -1.93 |
| GO:0007219 | Notch signaling pathway | 8 | 4.68 | -4.55 | -1.91 |
| GO:0010951 | negative regulation of endopeptidase activity | 9 | 5.26 | -4.50 | -1.88 |
| GO:0034113 | heterotypic cell-cell adhesion | 5 | 2.92 | -4.37 | -1.81 |
| R-HSA-8957275 | Post-translational protein phosphorylation | 6 | 3.51 | -4.26 | -1.72 |
| GO:0010812 | negative regulation of cell-substrate adhesion | 5 | 2.92 | -4.21 | -1.69 |

**Sorafenib**

| GO | Description | Count | % | Log10(P) | Log10(q) |
| --- | --- | --- | --- | --- | --- |
| GO:0002366 | leukocyte activation involved in immune response | 34 | 14.05 | -15.08 | -10.96 |
| GO:0030155 | regulation of cell adhesion | 27 | 11.16 | -9.48 | -6.28 |
| GO:0002521 | leukocyte differentiation | 21 | 8.68 | -7.94 | -4.80 |
| GO:0050900 | leukocyte migration | 19 | 7.85 | -6.82 | -3.73 |
| GO:0009617 | response to bacterium | 22 | 9.09 | -6.11 | -3.09 |
| GO:0033632 | regulation of cell-cell adhesion mediated by integrin | 4 | 1.65 | -5.77 | -2.80 |
| GO:0042116 | macrophage activation | 8 | 3.31 | -5.55 | -2.60 |
| GO:0070997 | neuron death | 14 | 5.79 | -5.46 | -2.53 |
| GO:0007229 | integrin-mediated signaling pathway | 8 | 3.31 | -5.27 | -2.42 |
| GO:0090087 | regulation of peptide transport | 19 | 7.85 | -5.09 | -2.32 |
| GO:0007249 | I-kappaB kinase/NF-kappaB signaling | 12 | 4.96 | -5.09 | -2.32 |
| GO:0110110 | positive regulation of animal organ morphogenesis | 7 | 2.89 | -5.04 | -2.29 |
| GO:0034109 | homotypic cell-cell adhesion | 7 | 2.89 | -4.97 | -2.25 |
| GO:0048663 | neuron fate commitment | 6 | 2.48 | -4.60 | -2.01 |
| GO:0050808 | synapse organization | 14 | 5.79 | -4.56 | -1.98 |
| GO:0061061 | muscle structure development | 18 | 7.44 | -4.51 | -1.95 |
| GO:0045089 | positive regulation of innate immune response | 13 | 5.37 | -4.43 | -1.92 |
| GO:0032496 | response to lipopolysaccharide | 12 | 4.96 | -4.42 | -1.92 |
| GO:0030036 | actin cytoskeleton organization | 18 | 7.44 | -4.31 | -1.83 |
| GO:0034112 | positive regulation of homotypic cell-cell adhesion | 3 | 1.24 | -4.29 | -1.82 |
